# Metabolomics based predictive classifier for early detection of pancreatic ductal adenocarcinoma

**DOI:** 10.18632/oncotarget.25212

**Published:** 2018-05-01

**Authors:** Keith Unger, Khyati Y. Mehta, Prabhjit Kaur, Yiwen Wang, Smrithi S. Menon, Shreyans K. Jain, Rose A. Moonjelly, Shubhankar Suman, Kamal Datta, Rajbir Singh, Paul Fogel, Amrita K. Cheema

**Affiliations:** ^1^ MedStar Georgetown University Hospital, Washington, DC, United States of America; ^2^ Department of Oncology, Lombardi Comprehensive Cancer Center, Georgetown University Medical Center, Washington, DC, United States of America; ^3^ Department of Biostatistics and Biomathematics, Georgetown University Medical Center, Washington, DC, United States of America; ^4^ Departments of Biochemistry and Molecular and Cellular Biology, Georgetown University Medical Center, Washington, DC, United States of America; ^5^ Unité MéDIAN, UMR CNRS 6237 MEDYC, Université de Reims, Reims, France

**Keywords:** PDAC, tissue metabolomics, predictive biomarkers

## Abstract

The availability of robust classification algorithms for the identification of high risk individuals with resectable disease is critical to improving early detection strategies and ultimately increasing survival rates in PC. We leveraged high quality biospecimens with extensive clinical annotations from patients that received treatment at the Medstar-Georgetown University hospital. We used a high resolution mass spectrometry based global tissue profiling approach in conjunction with multivariate analysis for developing a classification algorithm that would predict early stage PC with high accuracy. The candidate biomarkers were annotated using tandem mass spectrometry. We delineated a six metabolite panel that could discriminate early stage PDAC from benign pancreatic disease with >95% accuracy of classification (Specificity = 0.85, Sensitivity = 0.9). Subsequently, we used multiple reaction monitoring mass spectrometry for evaluation of this panel in plasma samples obtained from the same patients. The pattern of expression of these metabolites in plasma was found to be discordant as compared to that in tissue. Taken together, our results show the value of using a metabolomics approach for developing highly predictive panels for classification of early stage PDAC. Future investigations will likely lead to the development of validated biomarker panels with potential for clinical translation in conjunction with CA-19-9 and/or other biomarkers.

## INTRODUCTION

Pancreatic ductal adenocarcinoma (PDAC) represents 90% of pancreatic neoplasms and the fourth leading cause of cancer death in the United States [1, 2]. Due to the retroperitoneal location of the pancreas, these tumors are difficult to detect; moreover progression of pancreatic cancer is often asymptomatic until late stages of the disease [3–7]. Surgical resection offers the only opportunity for cure, however, since early diagnosis is uncommon, only 20% of patients are candidates for surgery while the majority of patients present with advanced disease. The median survival for advanced or metastatic pancreatic cancer is less than 5% at 5 years [8]. In contrast, the subsets of patients diagnosed with stage I disease or incidentally discovered PDAC have improved survival relative to symptomatic surgical patients [9]. Therefore, early detection is likely to improve outcomes.

Interestingly, classic mutations that are highly prevalent in PDAC such as the KRAS, TP53, SMAD4, and CDKN2A are known to regulate signaling pathways that impact central metabolic processes [10, 11]. Moreover, the unique physiology of pancreatic tumor microenvironment (TME) comprises a dense stroma that facilitates tumor growth despite high oxidative stress, inflammation, vasculature through metabolic adaptation [12, 13]. The TME creates a metabolically favorable niche for tumor cell proliferation and migration, by maintaining a highly immunosuppressive environment, which is partly regulated by metabolic alterations [14–16]. Taken together, the strong association of metabolic perturbations with pancreatic cancer pathophysiology makes a strong case for identifying metabolic changes that could then be used as specific biomarkers. In addition, this information could be used for identifying molecular targets that are druggable and thus actionable [17, 18].

Although several biomarker studies using “omics” approaches have been reported, they are yet to yield a reliable signature that could be used in the clinic to drive PDAC treatment decisions [19–34]. Carbohydrate antigen 19-9 (CA19-9) is the only biomarker in clinical use and is primarily indicated for monitoring response to therapy or recurrence of disease. In a pooled analysis, CA 19-9 was found to have a sensitivity of 79% and specificity of 82% [35]. CA 19-9 levels are affected by a variety of other conditions, including obstructive jaundice, pancreatitis, and inflammatory diseases. Mass screenings conducted in Japan and Korea in the 1980s with CA 19-9 and ultrasound were determined to be ineffective for detecting cancer in asymptomatic individuals [36, 37]. Although imaging modalities are useful in diagnosing pancreatic cancer in symptomatic individuals, these tests are limited by their invasive nature, lack of effectiveness, and cost, and do not have a role in screening asymptomatic patients [38].

Pancreatic cancer tumorigenesis is a prolonged process, requiring at least a decade to develop invasive pancreatic ductal adenocarcinoma [39]. This time window offers an opportunity for early detection of pancreatic cancer prior to the development of advanced or metastatic disease. Since PDAC is a relatively low prevalence cancer (10/100,000 individuals in US), the underlying goal for developing a predictive or an early detection panel for PDAC is to augment screening of high risk cohorts rather than the general population. Precursor lesions (PLs) to pancreatic cancer include pancreatic cystic lesions and pancreatic intra-epithelial neoplasia (PanIN) [40]. Two well described PLs which form pancreatic cysts are intraductal papillary mucinous cystic neoplasms (IPMNs) and mucinous cystic neoplasms (MCNs). Cystic neoplasms with high risk features for progression to or association with malignancy are considered for resection [41]. Non-invasive PLs of the pancreas can give rise to invasive pancreatic carcinoma over a relatively long lag time during which patients often remain asymptomatic. Molecular profiling of pancreatic tissue obtained from patients with benign pancreatic disease, pre-malignant or malignant lesions of the pancreas, offers an information rich matrix for discovering specific bio-signatures with potential application for early detection of pancreatic cancer whose performance can then be evaluated in plasma for developing minimally invasive assays.

Thus, the overall goal of this study was to identify specific metabolite signatures in tissue that are highly associated with precursor pancreatic lesions and early stage invasive pancreatic cancer as compared to the benign group. We hypothesized that certain tissue metabolite profiles would be shared between early stage pancreatic cancer patients (stages IA, IB, IIA) and the high risk cohort (PL) when compared to the benign group and the disease control group (colorectal cancer (CRC)). We further posited that targeted, quantitative blood based evaluation of molecular fingerprints of early stage pancreatic cancer, calculated originally via matched tissue metabolomics/lipidomic profiles, using an untargeted approach, may have direct clinical applicability.

Therefore, we used high resolution mass spectrometry based metabolomics, an emerging field that provides new information on biological perturbations based on changes in abundance of multiple endogenous metabolites. Since endogenous metabolism represents the endpoint of cellular processes and are hence a direct readout of the phenotype or the physiological status. Technological advances in mass spectrometry (MS), in combination with multivariate statistical methods provide a promising approach for developing molecular fingerprints of the diseased state.

**Figure 1:**
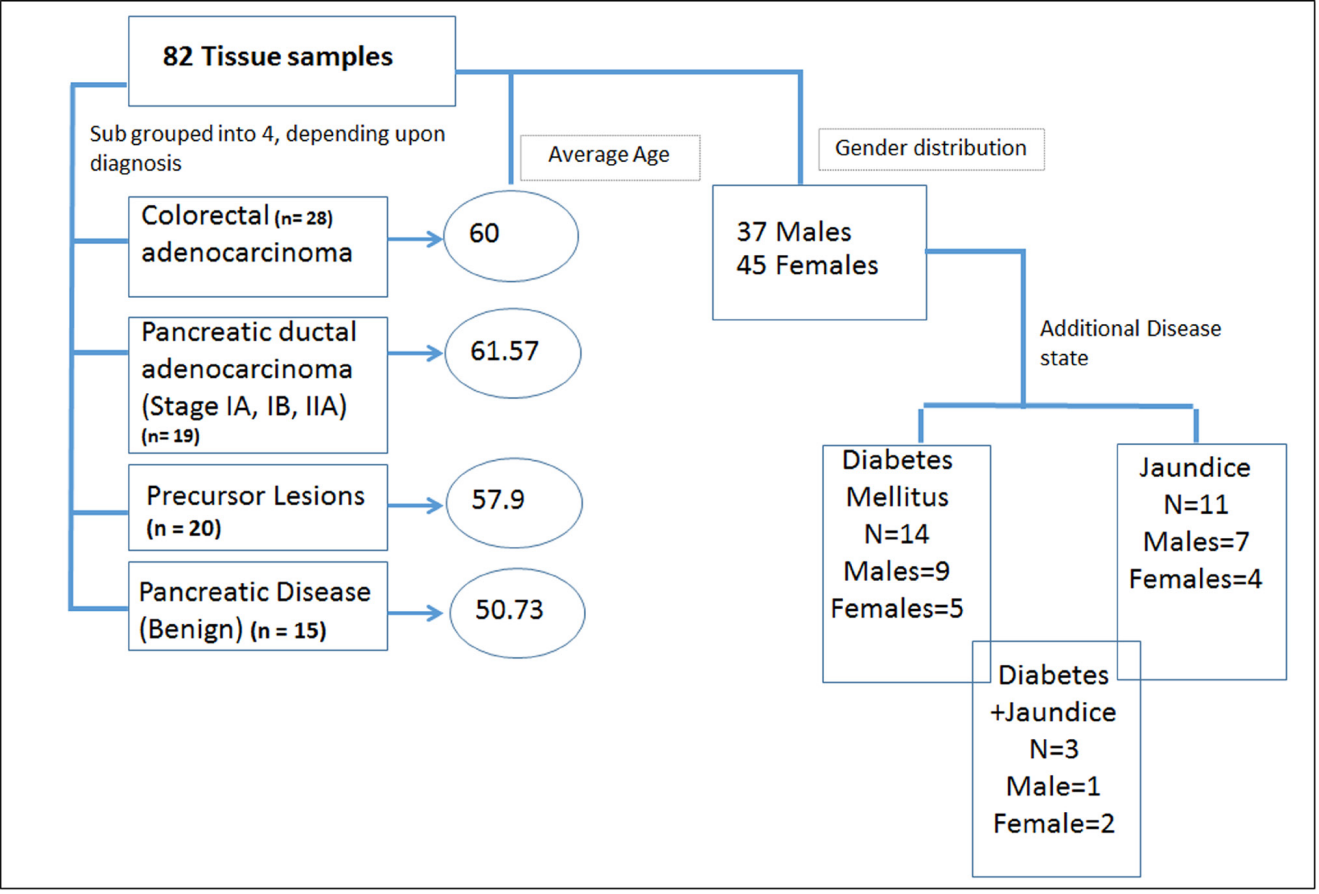
Demographic and clinical characteristics of the cohort used for metabolomics analyses

Comparative metabolomics profiling was performed for tissue derived from patients diagnosed with benign pancreatic diseases (benign, *n* = 15), patients diagnosed with cysts with possible malignant potential, representing a high risk cohort for pancreatic cancer (PL group, *n* = 20) and surgically excised tumor tissue from patients that were diagnosed with pancreatic cancer (early stage PDAC group, *n* = 19). Tumor tissue from patients with invasive adenocarcinoma of the colon (CRC group, *n* = 28) was used as a cancer disease control group. The tissue and matched plasma samples were obtained from a clinical cohort that received treatment at MedStar Georgetown University Hospital and banked in the Indivumend bio-repository (Figure 1). A shortlisted panel of these putative markers was annotated in tissue and subsequently evaluated in plasma samples obtained from the same patients using targeted mass spectrometry. The underlying idea of this combinatorial approach was to interrogate the tissue metabolome in a global and unbiased fashion and subsequently test the clinical applicability using a targeted approach in matched plasma samples. Tissue metabolomics yielded several metabolites that showed dysregulation in early stage PDAC when compared to benign or CRC groups. A panel of six metabolites resulted in a classifier that could stratify early stage PDAC and benign cases with >95% accuracy (AUC = 0.95), while the same classifier had a lower predictive value for classifying CRC cases, thus emphasizing the specificity of the biomarker panel. These findings demonstrate the feasibility of developing early detection panels for accurate classification of PDAC; however, when tested in pre-malignant cases, this panel demonstrated diminished efficacy.

## RESULTS

### Clinical cohort characteristics

We leveraged pristinely collected and extensively annotated post-surgical samples form Indivumed repository at the Georgetown University Hospital. The clinical cohort used in this study consisted of a local population of patients with clinical presentation of benign (*n* = 15), precancerous lesions (PL) (*n* = 20) or early stage (IA, IB, IIA) PDAC (*n* = 19). The benign pancreatic disease group included patients with a clinical diagnosis of pancreatitis and pancreatic cystic neoplasms that have a benign natural history. While a history of pancreatitis is a known risk factor for the development of PDAC, pancreatitis is not a neoplastic process. Pancreatic cystic neoplasm included in the benign pancreatic disease cohort, such as serous cystadenomas, are almost never malignant and do not require imaging frequent surveillance in clinical practice. PL included patients who underwent surgical resection for IPMN (*n* = 14), MUC (*n* = 3), or other (*n* = 3) and were judged to be at high risk for progression or association of invasive disease. Criteria for resection was based on commonly used consensus clinical practice guidelines [42]. Although PLs exhibit a spectrum of clinical behaviors, this patient cohort was enriched for cystic pancreatic neoplasms with clinical, radiologic, and/or cytologic features associated with progression to invasive pancreatic cancer. CRC was used as a disease control group to test biomarker specificity (*n* = 28). The clinical characteristics are detailed in Supplementary Table 1. The average age of the cohort was 58.6 years with almost uniform gender distribution. A total of 14 patients including six PDAC and five benign cases also had a diagnosis of Type II or Type I diabetes mellitus (DM), while a total of 11 patients had jaundice as a co-morbidity in benign (*n* = 2) and PDAC (*n* = 9) (Figure 1 and Supplementary Table 1). The PDAC, benign and PL cohorts did not receive any chemotherapy or radiation treatment prior to surgery.

### Predictive biomarkers of early stage PDAC

**Figure 2:**
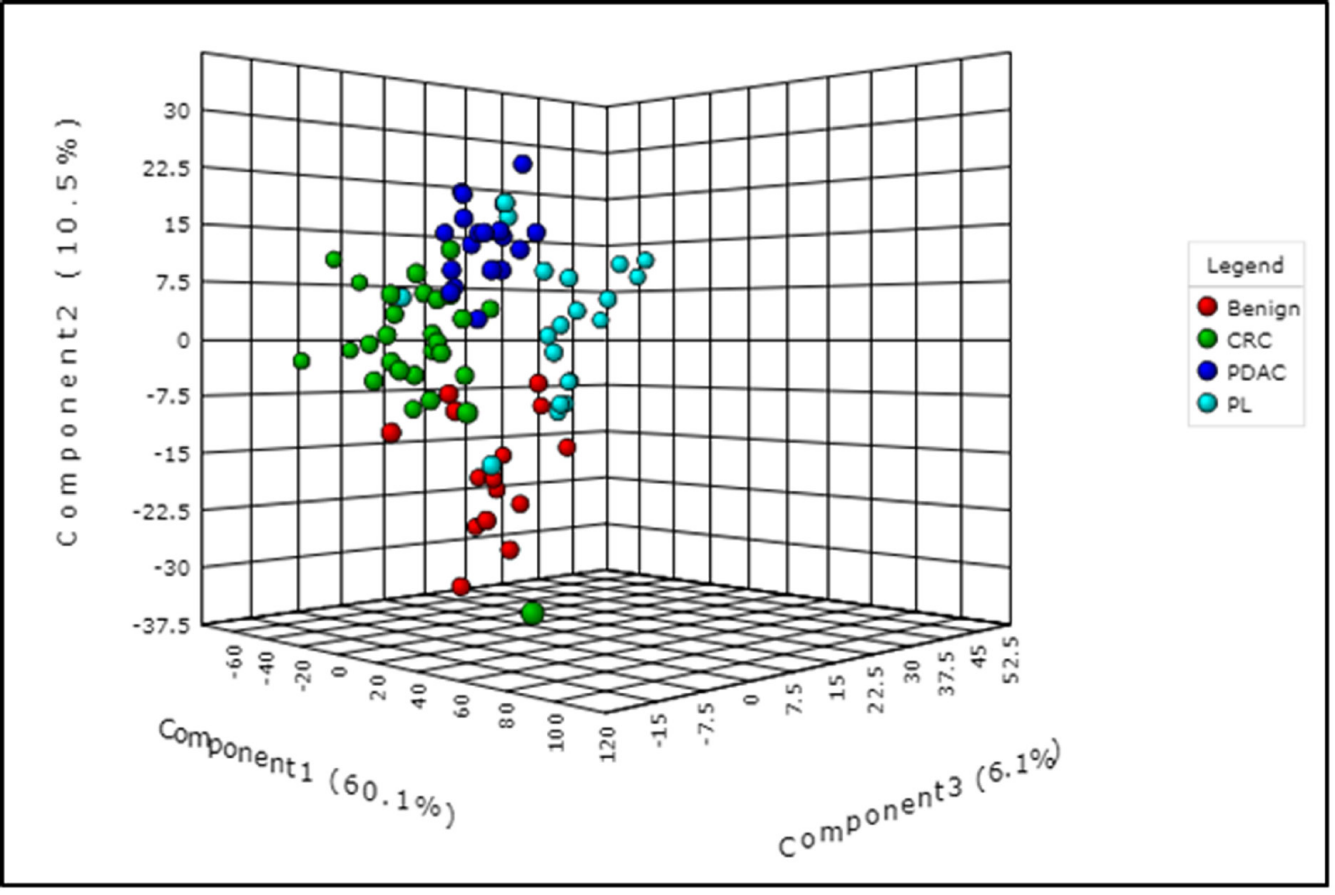
Partial least squares discriminant analysis (PLS-DA) plot showing interclass separation between the different diagnostic groups (pancreatic disease (benign), colorectal cancer (CRC), pancreatic lesions (PL), and pancreatic ductal adenocarcinoma (PDAC), based on overall tissue metabolite profile

**Figure 3:**
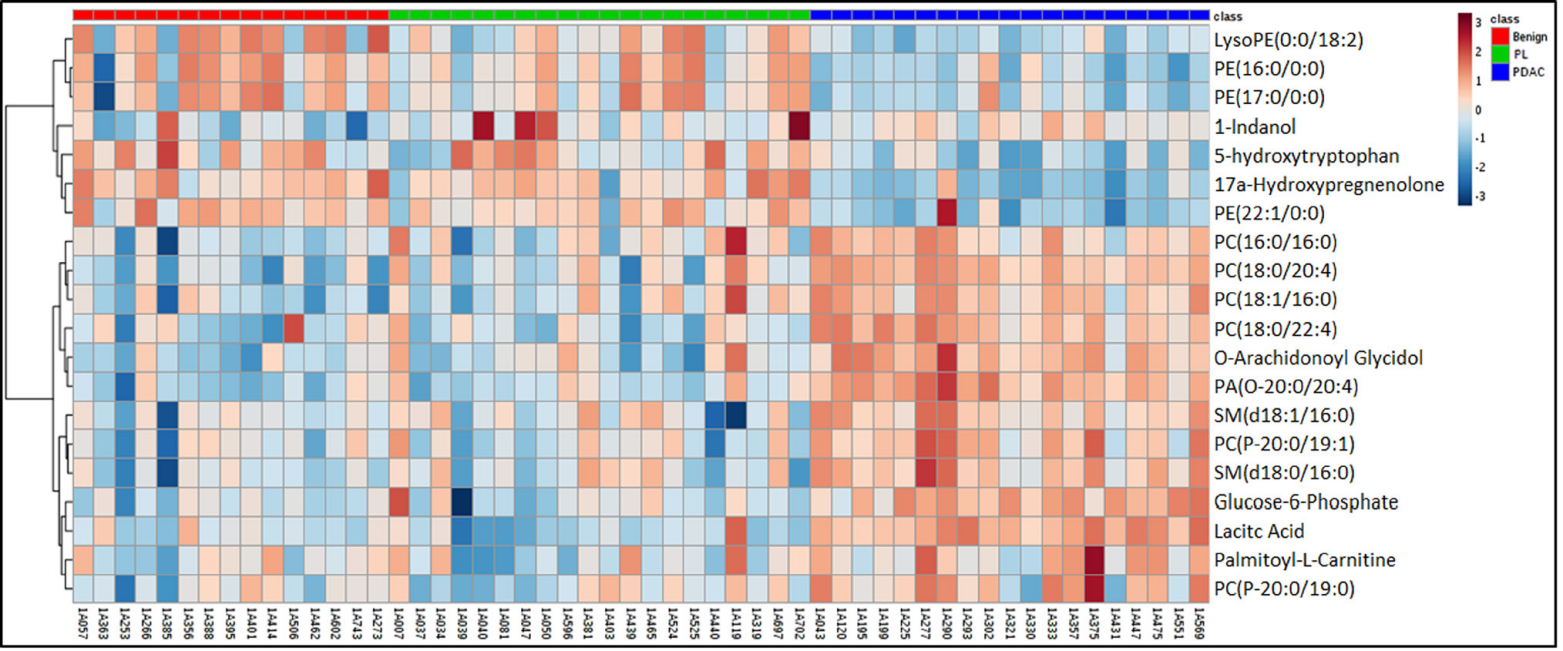
Heat map illustration of dysregulated metabolites in pancreatic lesions (PL) and pancreatic ductal adenocarcinoma (PDAC) gro ups as compared to the benign pancreatic disease group

As described above, we used pancreatic tissue as the most direct and specific matrix for biomarker identification, as it is likely to have the highest concentrations of disease specific markers. We have previously reported on the feasibility of this approach for discerning biomarkers of PDAC [43]. We used a high resolution mass spectrometry based untargeted metabolomics profiling approach to delineate candidate markers that would augment stratification of these diagnostic groups. UPLC-ESI-TOF-MS analysis in the aqueous extract (AE) yielded 1532 features in the positive mode and 1866 in the negative mode. The organic extract (OE) yielded 1277 features in the positive mode and 1358 features in the negative mode. We selected the positive mode OE as a representative dataset for data visualization using a partial least squares discriminate analysis (PLS-DA) model (Metaboanalyst v3.0) for discriminating PDAC, PL, CRC and benign groups (Figure 2) with a 5 component R^2^ value of 0.78 and Q^2^ value of 0.45. The four diagnostic groups showed maximum separation along two components (Component 1 = 60.1% and Component 2 = 10.5%) providing good support to the model. The CRC and the PDAC showed a clear group separation, while the PL group clustered between the benign and the PDAC groups. In addition, a PLS-DA plot was generated for the data including the quality control samples (Supplementary Figure 1) with a 5 component R^2^ value of 0.87 and Q^2^ value of 0.66 to show a good clustering of QC, which demonstrates minimal variability during the run. Students *T*-test was used to identify 379 candidate biomarkers between benign, PDAC, and PL of which 85 were annotated using tandem mass spectrometry. R^2^ and Q^2^ values for the binary comparison of the representative data (OE positive mode) were also generated (Supplementary Table 2). Remarkably, lipids were found to be the pre-dominant class of metabolites that showed differential abundance in the PL and PDAC groups as compared to the benign group (Figure 3) and in the CRC and PDAC groups as compared to the benign group (Supplementary Figure 2). Pathway analysis for metabolites found dysregulated in PDAC and CRC (as compared to the benign group), was performed to identify biochemical perturbations that are shared between the two malignancies (Supplementary Figure 3). Our results showed that metabolites associated with inflammation, phospholipid metabolism, Ca^2+^ flux, and cell junction signaling pathways were impacted in CRC and PDAC.

**Figure 4:**
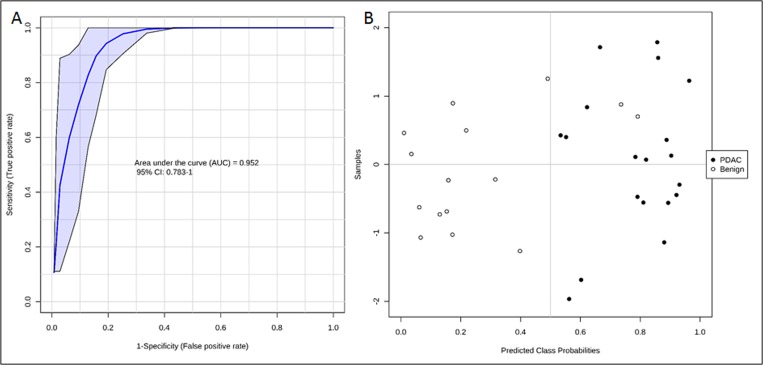
ROC curve (**A**) and predicted class probabilities for six metabolite panel (**B**) showing high classification accuracy between PDAC and Benign.

**Figure 5:**
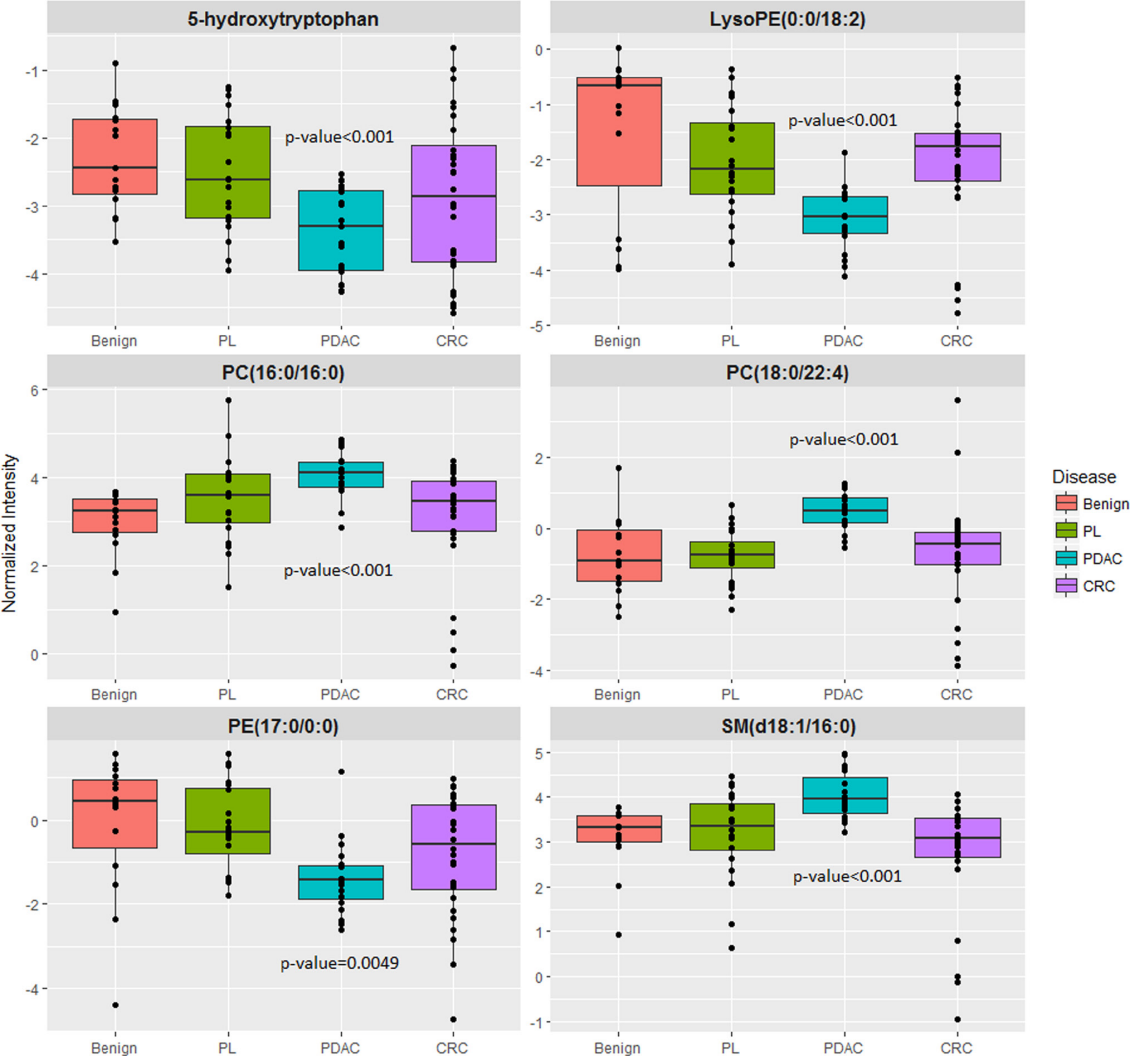
Six metabolite panel shows a shared pattern in the PL and PDAC group as compared to benign

Following feature selection of significantly dysregulated metabolites between PDAC and benign pancreatic disease group (Supplementary Table 3), we used the SVM based ROC analysis (Metaboanalyst 3.0), for developing robust classification algorithms, predictive of early stage pancreatic cancer with high accuracy. This led to the development of a six metabolite panel that could distinguish benign cases from those with early stage PDAC, with high accuracy (AUC = 0.95, SP = 0.85 and SN = 0.9) (Figure 4). The six metabolite panel consisted of 5-hydroxytryptophan, LysoPE(18:2), PC(16:0/16:0), PC(18:0/22:4), PE(17:0), and SM(d18:1/16:0) (Table 1). These metabolites are found to be significantly associated with early stage PDAC as compared to the benign pancreatic disease group. Furthermore, each of the metabolites showed a linear trend comparing from the benign to the PL and finally to the PDAC groups (Figure 5), implicating their role in defining disease progression. This change was not significant in the CRC group (AUC = 0.45) (Supplementary Figure 4), thus establishing specificity for the PDAC group. We used this panel construct a ROC curve to compare the PL and benign groups. This resulted in an AUC of 0.462 (Supplementary Figure 5), suggesting that classifiers built with early stage diagnostic groups of PDAC did not perform with the same efficacy in the high risk cohort with pre-malignant lesions.

**Table 1:** Six metabolite panel performance measures across different comparative groups

		PDAC		PL		CRC	
Metabolite name	m/z	Fold change (PDAC/Benign)	*p*-value	Fold change (PL/Benign)	*p*-value	Fold change (CRC/Benign)	*p*-value
5-hydroxytryptophan	221.0332	↓ 0.44	7.85e-5	0.89	0.46	0.81	0.1
LysoPE (0:0/18:2)	478.2947	↓ 0.24	1.27e-4	0.6	0.2	0.58	0.14
PC(16:0/16:0)	734.5696	↑ 2.09	2.51e-5	1.68	0.08	1.24	0.82
PC(18:0/22:4)	838.6341	↑ 1.93	9.35e-5	0.84	0.96	1.56	0.87
PE (17:0/0:0)	466.2952	↓ 0.33	0.0049	0.81	0.84	0.58	0.12
SM(d18:1/16:0)	703.574	↑ 1.92	1.16e-4	1.18	0.78	0.93	0.37

### There is a misalignment of markers in tissue and blood

Since our goal was to delineate a specific metabolite panel using tissue metabolomics followed by validation in matched plasma samples, we next used multiple reaction monitoring mass spectrometry for targeted quantification of the 6 metabolites in plasma samples obtained from the same set of patients diagnosed with benign pancreatic disease group, PL or early stage PDAC. As discussed previously, CRC was used as a disease control group to test biomarker specificity. Of the six metabolites we obtained reproducible results for quantitative evaluation of three metabolites PC(16:0/16:0), PC(18:0/22:4), and SM(d18:1/16:0) while the PE(17:0) levels in the plasma samples were lower than the lower limit of quantification (LOQ). Further, we found that the relative abundance in plasma for the assayed metabolites in the respective comparative groups was not concordant with that observed in tissue, for most part (Supplementary Table 4).

### Shared metabolite expression patterns in cancer

We leveraged the inclusion of CRC in our study design to identify metabolites that exhibit a common trend in the two malignancies as compared to the benign group. Our analyses helped delineate several metabolites that showed significant differences between benign cases as compared to the PDAC as well as in the CRC cohort (Supplementary Table 5). This included a decrease in tissue levels of arachidonic acid and eicosanoids in tumor tissue, with a concomitant increase in prostaglandin E2 (Supplementary Figure 6). We also observed an increase in Cox 2 staining of the pancreatic tumor tissue as compared to matched normal tissue which was suggestive of diversion of arachidonic acid pathway towards the production of pro-inflammatory mediators known to be associated with malignant progression (Supplementary Figure 7). Seven metabolites were found to be dysregulated for the PDAC, CRC, and PL groups (Supplementary Table 6). In addition, we identified metabolites that were shared in PDAC and PL tissue samples as compared to the benign groups (Supplementary Table 7) and those found uniquely significant in PL (Supplementary Table 8) could be used for pre-malignant classification. ROC analysis using these significantly dysregulated metabolites yielded an AUC of 0.836 for a 12 metabolite panel for classifying PL from benign using a metabolite fingerprinting approach since the identities of the metabolites was unknown when searched against publicly available data bases. (Supplementary Figure 8).

## DISCUSSION

Pancreatic cancer is rarely detected when the disease is localized and amenable to curative treatment, representing a major challenge to improving outcomes. Over two thirds of patients with PDAC are initially diagnosed with advanced disease (stage III or IV) at presentation. Advanced disease is associated with poor outcomes, with a median survival of less than 1 year with few patients alive at 5 years [44]. Mutational analysis suggests that the development of metastatic disease is a late event, requiring at least 10 years, thus providing a window of time for early detection [39]. However, early detection of pancreatic cancer remains a major barrier towards improving clinical outcomes of the disease. A worldwide expert consortium has advocated for pancreatic cancer screening strategies aimed at detecting asymptomatic precursor lesions and early stage pancreatic cancer as a means to improving outcomes [45]. Molecular based phenotyping approaches have great potential to aid in early detection efforts for curable lesions of the pancreas [46].

We leveraged the unique clinical characteristics of our clinical cohort to delineate molecular patterns that were associated with early stage pancreatic cancer and precursor pancreatic lesions that were amenable to potentially curable surgical resections. A colorectal cancer cohort was used as a cancer disease control group to eliminate non-specific biomarkers. CRC was selected as a cohort because the histology is similar to PDAC and both cancers arise in the gastrointestinal track. Benign pancreatic diseases include clinical entities with no malignant potential or patients with benign pancreatic disease group, an inflammatory condition of the pancreas. Several previous studies have used healthy controls to identify biomarkers associated with PDAC [47–49]. However existing biomarkers, including CA19-9 are often elevated in the setting of benign pancreatic diseases, limiting their clinical utility [50]. By developing biomarkers which are able to differentiate PDAC from benign pancreatic disease, this approach could offer improved specificity.

We used high resolution mass spectrometry based untargeted metabolomic profiling of tissue to identify and quantify candidate biomarkers that could discriminate early stage PDAC from the benign cohort and CRC with high accuracy. A six metabolite panel was developed that accurately distinguished PDAC from benign pancreatic diseases in pancreatic tissue (AUC = 0.95). There was a linear trend when comparing the relative intensities of metabolites going from the benign group to the PL group and finally to the PDAC group, suggesting a role for these metabolites in progression to invasive carcinoma. The six metabolite biomarker panel did not yield high predictive power for classification of the CRC group (AUC = .45), emphasizing specificity for classification of early stage PDAC. Additionally, this panel did not discriminate the PL from the benign group (AUC = 0.46), suggesting that a biomarker panel developed for early stage PDAC group may not demonstrate the same accuracy when tested for pre-malignant classification. This underscores the need for studies that use pre-diagnostic biospecimens for development of classification algorithms that would help predict early onset of PDAC in clinically asymptomatic patients.

The six metabolite panel consisted of LysoPE (0:0/18:2), PC(16:0/16:0), PC(18:0/22:4), PE (17:0/0:0), SM(d18:1/16:0) and 5-hydroxytryptophan. Several groups have reported the importance of lipids as powerful discriminators between PDAC and normal controls [51], [18]. 5-hydroxytryptophan is an intermediate in serotonin biosynthesis; decreased levels in PL and PDAC could reflect increased conversion to serotonin which has been implicated in tumor growth and progression [52, 53]. Taken together, our results show markers that can be early markers of disease onset and progression and need further validation for exploring potential clinical utility.

Additionally, we found that several metabolites significantly differed between the PDAC and CRC groups as compared to the benign group. Pathway analysis suggested dysregulation of metabolites mediating inflammation, Ca^2+^ flux and cell junction signaling, that may augment cell proliferation, invasion and angiogenesis which are known hallmarks of carcinogenesis [54]. These changes suggest that there are underlying commonalities associated with malignancy which could be useful as a pan-cancer approach wherein delineation of shared molecular patterns helps detect cancer, instead of organ of origin [55].

Next, we used targeted mass spectrometry for quantification of these six metabolites in plasma samples from the same cohort. We found that although some of these metabolites were detectable in plasma samples with a high signal to noise ratio, the relative abundance for most metabolites in plasma were not concordant with results from the discovery experiments performed with tissue samples. These findings emphasize that although tissue is a specific matrix for interrogating local metabolic changes in tumor, the findings cannot always be extrapolated directly in blood, possibly because the signal gets averaged out in circulation. More recently, studies from other laboratories have also shown that there is a misalignment of markers between tissue and blood [56]. While tissue is an information rich matrix to understand molecular underpinnings that define local disease progression, bio-fluids are matrices of choice for developing minimally invasive, biomarker panels that could be directly used for large scale validation studies with diverse cohorts to test clinical utility.

Due to the relative low prevalence of pancreatic cancer in the general population, widespread screening may not be practical. An effective early detection program will likely require identification of individuals at significantly elevated risk. Individuals at greater than 5% lifetime risk for developing PDAC include those with a family history of PDAC and/or germline mutations carriers which have been associated with significantly increased risks for PDAC [57]. Other non-genetic risks factors including active tobacco abuse, heavy alcohol use, obesity, and diabetes can be used to help identify additional individuals at higher than average risk of pancreatic cancer [58]. Although we were able to delineate a specific panel with the tissue specimens, implementation of this panel in the clinic would require invasive sampling procedures like tissue biopsy. Thus this method would not be ideal as a screening test even for high risk individuals since the prevalence of pancreatic cancer is low (10/100,000). Furthermore, the overall goal was to validate this panel in matched plasma samples to develop a minimally invasive assay; however we found the metabolite abundance to be discordant. Hence, ongoing investigations from our laboratory will focus on developing plasma/serum based biomarker panels that after suitable external validation could be used for clinical efficacy studies.

## METHODS

### Sample collection

Collection of the biospecimens was approved by MedStar Georgetown University Hospital IRB and all patients signed informed consent. The tissue samples (*n* = 82) were obtained from the Indivumed repository at Georgetown University and are pristinely collected with extensive clinical annotations. Samples were obtained under fasting conditions of at least 12 hours. The samples were further classified into 4 subgroups: benign, precursor lesions (PL), CRC, and PDAC. The clinical details are summarized in Figure 1. These samples were collected using very stringent and consistent protocols for collection, samples processing and storage. All banked plasma samples were collected between 2010 and 2015 and stored at –80° C and underwent one freeze thaw before analysis. We have checked inter-day variability for these metabolites on a QTOF instrument and determined that the signal drops on the fifth day when samples are in the autosampler. When stored at –80° C, the metabolites are stable over time.

### Metabolite extraction

Sample preparation schema is detailed in Supplementary Figure 9 which is a slightly modified version of the protocol described by Want *et al.* [59]. Briefly, tissue samples (100 micron sections) were homogenized using Powergen-125 (Roche), for three cycles of 30 seconds each with 500 µL of prechilled 50% methanol containing internal standards (10 µL of debrisoquine and 50 µL of 4-NBA (1 mg mL^−1^). The tissue samples were kept on ice during homogenization. The homogenized samples were centrifuged for 15 minutes at 4° C. The supernatant was carefully removed followed by protein precipitation which was performed by adding 100 µL of ACN, vortexed briefly and kept on ice for 20 minutes and centrifugation at 14 K for 15 minutes at 4° C. The supernatant was removed carefully vacuum dry, resuspended in 50% methanol and considered as aqueous extract. An aliquot of 2 µL from each sample was pooled together and used to condition the column and to run in between sample injections to test the sensitivity, retention time shifts or change in mass accuracy. The residual pellets after aqueous extraction were sonicated for 90 seconds with dichlorometahane in Methanol (3:1) containing internal standards (ceramide [5 pmole µL^−1^] and LPA 10 pmol µL^−1^) in glass vials. We used glass vials to avoid plastic contamination by dichloromethane or chloroform. The tissue homogenate was centrifuged at 14 K for 15 minutes at 4° C. The proteins were precipitated by adding 100 µL of ACN as described previously. The supernatant was dried under vacuum and resuspended in 85% methanol in IPA and considered as organic extract. An aliquot of 2 µL from each sample was pooled together to make the QC’s. The remaining solid pellet was used for the protein estimation for normalization of MS raw data.

### UPLC-TOFMS data acquisition

The data for aqueous and organic extracts were acquired separately. An aliquot of the aqueous extract (2 µL) from each sample was resolved on Acquity HSS T3 1.8µM, 2.1 × 100 mm column (Waters Corporation). A gradient of mobile phase consisting 100% methanol containing 0.1% formic acid (Solvent A), 100% water containing 0.1% formic acid (Solvent B) was resolved for 12 minutes at a flow rate of 0.4 ml/min. The data were acquired on an ESI-QTOF instrument (Xevo G2 QTOF, Waters Corp, USA) operating in either positive or negative electrospray ionization mode. The capillary voltage was set at 3.0 kV in positive mode and 2.8 in negative mode (another parameters were same in both the modes), sampling cone voltage was 30 V, source temperature was 120° C and desolation gas flow was 750 L h^−1^. Accurate mass was maintained by introduction of LockSpray interface of Leucine-enkephalin (556.2771 [M + H]^+^ or 554.2615 [M − H]^−^) at a concentration of 2 pg mL^−1^ in 50% aqueous acetonitrile and a rate of 2 ml min^−1^. Data were acquired in centroid mode from 50–1200 mass-to-charge ratio (*m/z*) in MS scanning.

The organic extract was resuspended in 85% methanol in IPA and resolved onto a reverse-phase 50 × 2.1 mm Acquity 1.7 µm BEH C18 column (Waters Corp.) using an Acquity UPLC system (Waters Corp.). A gradient of mobile phase comprising 100% H_2_O containing 0.1% formic acid (Solvent A), 100% ACN containing 0.1% formic acid (Solvent B) and 90% IPA in ACN containing 0.1% formic acid (Solvent C) was resolved for 13 minutes at a flow rate of 0.4 ml/min. The data were acquired on a G2-QTOF mass spectrometer operating in positive or negative electrospray ionization mode. The capillary voltage was set at 3.0 kV in positive mode and 2.8 in negative mode (another parameters were same in both the modes), sampling cone voltage was 30 V, source temperature was 120° C and desolvation gas flow was 750 L h^−1.^ Accurate mass was maintained by introduction of LockSpray interface of Leucine-enkephalin (556.2771 [M + H] ^+^or 554.2615 [M − H] ^−^) at a concentration of 2 pg mL^−1^ in 50% aqueous acetonitrile and a rate of 2 ml min^−1^. Data were acquired in centroid mode from 50–1200 mass-to-charge ratio (*m/z*) in MS scanning. The raw data files were converted into netCDF files using Masslynx and markers were extracted using XCMS (Scripps). The output intensity data were normalized to that of internal standards, as well as to the total protein concentration. Accurate mass based database search was performed to assign putative identifications to the peaks of interest. The identity of metabolites of interest was confirmed using tandem mass spectrometry (Supplementary Table 9). Quantitative analysis of metabolites in plasma samples was performed using stable isotope dilution-multiple reaction monitoring mass spectrometry using AbsoluteIDQ p180 kit (Biocrates, Innsbruck, Austria). The data were pre-processed using TargetLynx v3.0 (Waters Corporation, USA) and processed with the MetIDQ software (Biocrates). Statistical analyses were performed within the Metaboanalyst v3.0.

### Statistical analysis

Following data pre-processing and ion annotation, the initial abundance values of the measured metabolites were log transformed in order to stabilize variance, followed by pareto scaling to achieve empirical distribution of intensities across samples. Each data set from the two extracts and their respective ionization mode was treated independently. Differential expression between various patient groups was assessed using independent (unpaired) sample Student’s *t*-test. False discovery rate was controlled by the method of Benjamini and Hochberg at 5% significance level. Statistical analyses were performed using in-house R scripts while MetaboAnalyst v3.0 [60] was used to perform biomarker analyses and generate figures. After tandem mass spectrometry based validations, the significantly dysregulated metabolites from the 4 datasets were merged for biomarker analyses.

For selection of candidate biomarkers of early stage pancreatic cancer, we selected featured metabolites that best distinguished PDAC and patients with PLs, from those with benign pancreatic disease. CRC was used as a cancer disease control group to eliminate generic biomarkers of cancer, thus augmenting the selection of biomarkers that are specific to pancreatic cancer. The classification performance of the biomarker panel was assessed using the area under the ROC (receiver operating characteristic) curve (AUC). The ROC curve can be understood as a plot of the probability of classifying correctly the positive samples against the rate of incorrectly classifying true negative samples. We used biomarker analysis function of MetaboAnalyst v3.0 for plotting ROC curves and for predictive accuracy plots for all comparisons. ROC curves were generated by using Linear SVM as the classification method and with t-statistics as the feature ranking method. The procedure was repeated multiple times to calculate the performance and confidence interval of each model. Ingenuity pathway analysis tool was used for network analysis of metabolites that were commonly dysregulated in the two malignancies (CRC and PDAC) when compared to the benign pancreatic disease group.

## CONCLUSIONS

The overall goal of our study was to develop a non-invasive screening test for early stage pancreatic cancer in asymptomatic individuals who are potentially eligible for curative therapies. In conclusion, we delineated a panel of metabolites highly predictive for early stage classification of pancreatic cancer using tissue metabolomics. This study creates a foundation for future biomarker studies involving larger sample sizes in high risk individuals. This biomarker panel could then be used for increased surveillance of individuals deemed at high-risk of developing PDAC thus augmenting early detection.

## SUPPLEMENTARY MATERIALS FIGURES AND TABLES









## Abbreviations

PDAC: Pancreatic Ductal Adenocarcinoma
TME: Tumor Microenvironment
CA19-9: Carbohydrate antigen 19-9
PL: Precursor Lesion
IPMN: Intra-ductal Papillary Mucinous Neoplasm
MCN: Mucinous Cystic Neoplasia
PanIN: Pancreatic Intra-epithelial Neoplasia
CRC: Colorectal Cancer
MS: Mass Spectrometry
