# Supplementary material for: Metabolomics based predictive classifier for early detection of pancreatic ductal adenocarcinoma
Source: Oncotarget. 2018 May 1;9(33):23078–90. doi: 10.18632/oncotarget.25212 (PMC5955422; doi:10.18632/oncotarget.25212)
Supplement: Supplementary file 1 [file oncotarget-09-23078-s001.pdf]

Metabolomics based predictive classifier for early detection of pancreatic ductal adenocarcinoma

SUPPLEMENTARY MATERIALS

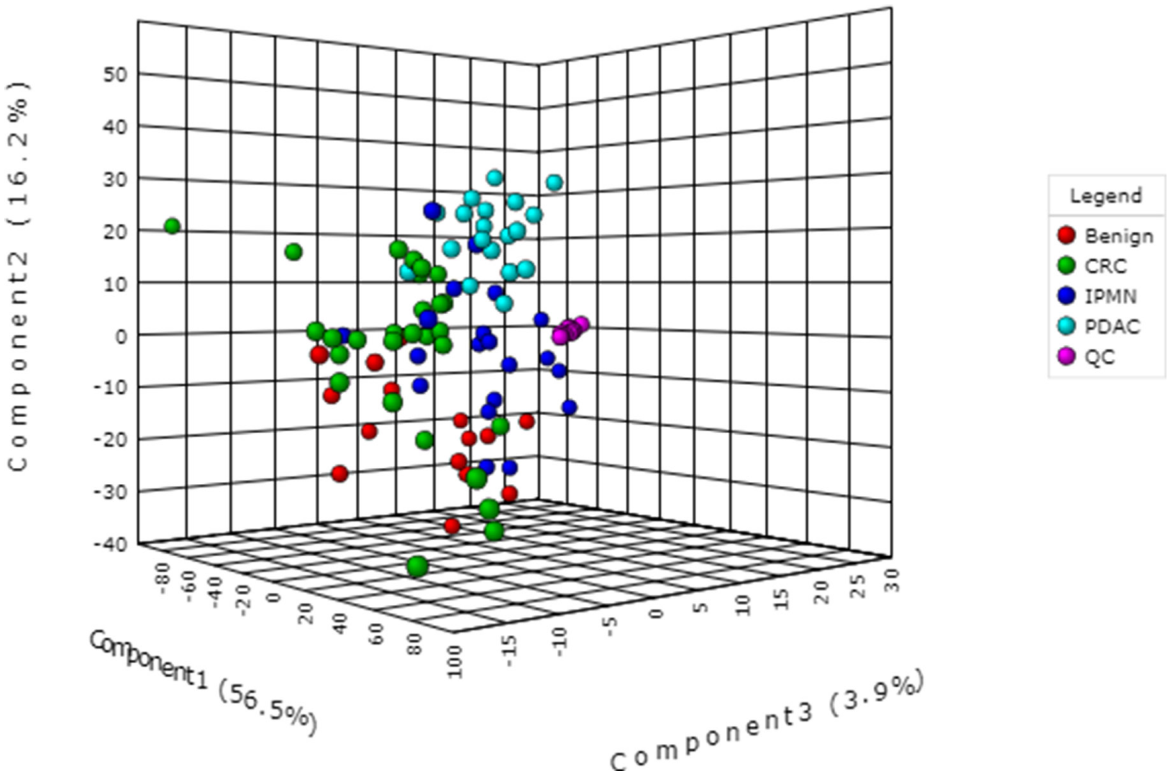

Supplementary Figure 1: Partial least squares discriminant analysis (PLS-DA) plot showing interclass separation between the different diagnostic groups and QC clustering.

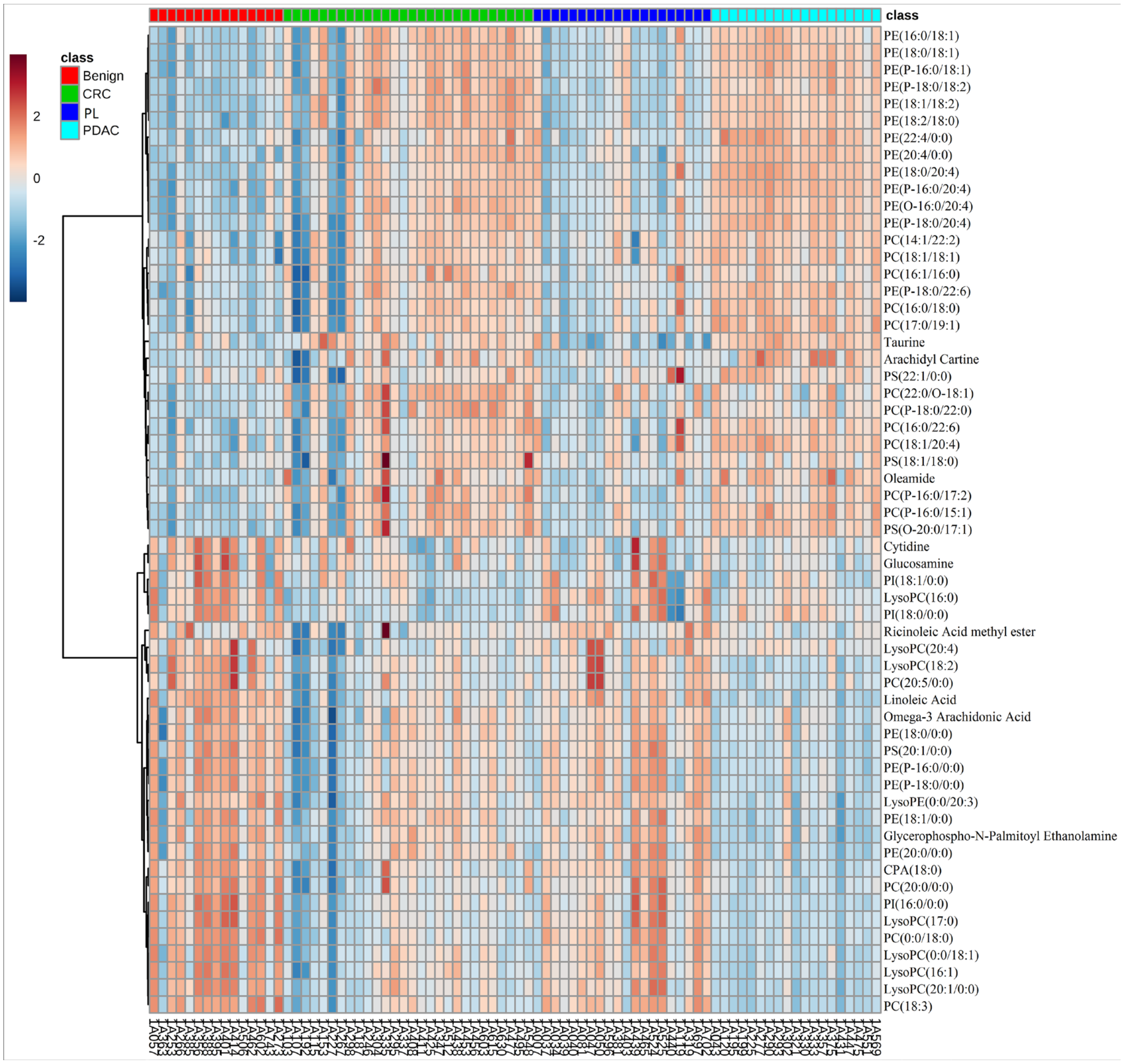

Supplementary Figure 2: Putative metabolite markers dysregulated in CRC and PDAC when compared to the benign pancreatic disease groups.

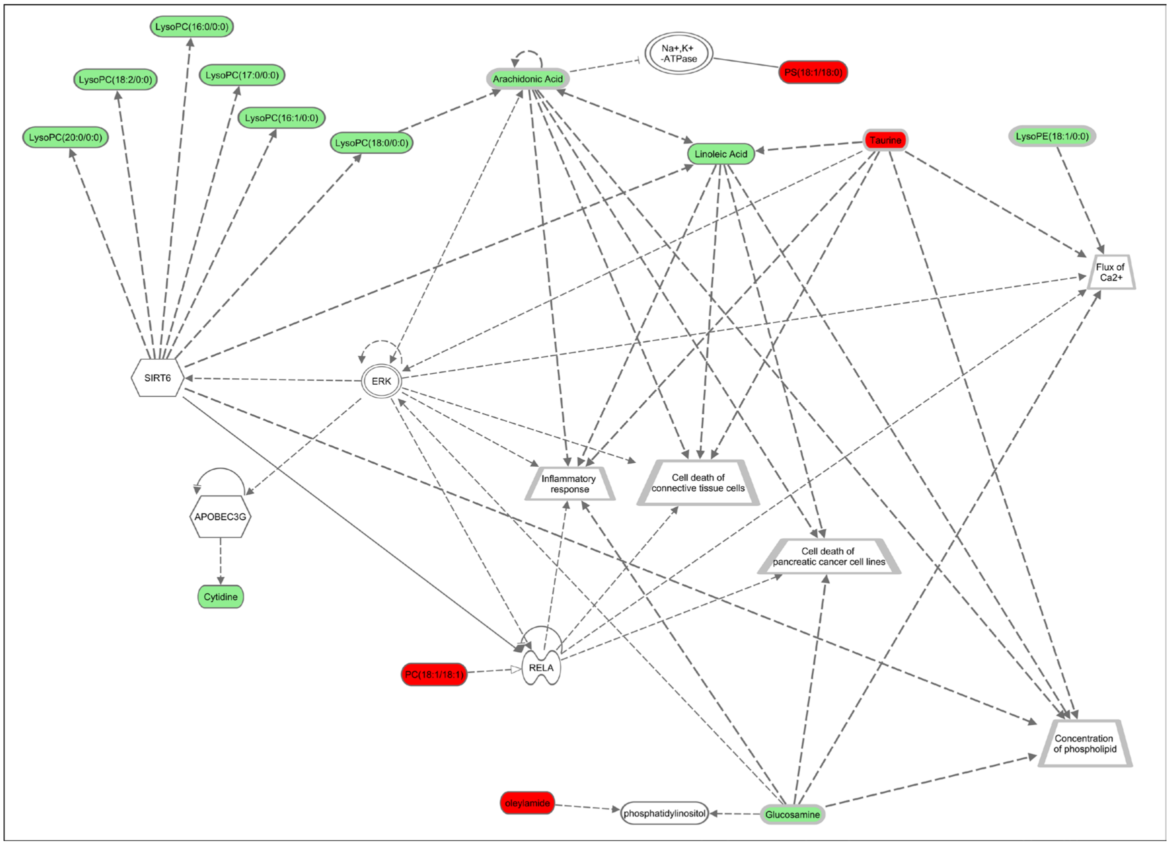

Supplementary Figure 3: Pathway analysis of common metabolites that were dysregulated in PDAC and CRC. Metabolites marked in red represent upregulation while green represent down regulation in both malignancies as compared to the benign group.

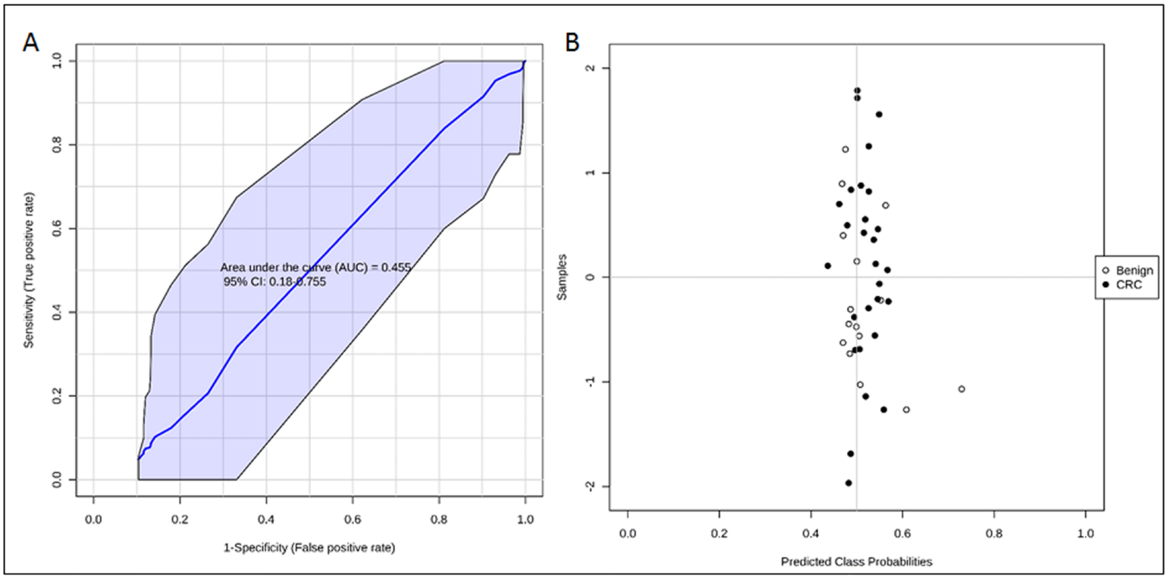

Supplementary Figure 4: ROC curve (**A**) and predicted class probabilities (**B**) between CRC and Benign.

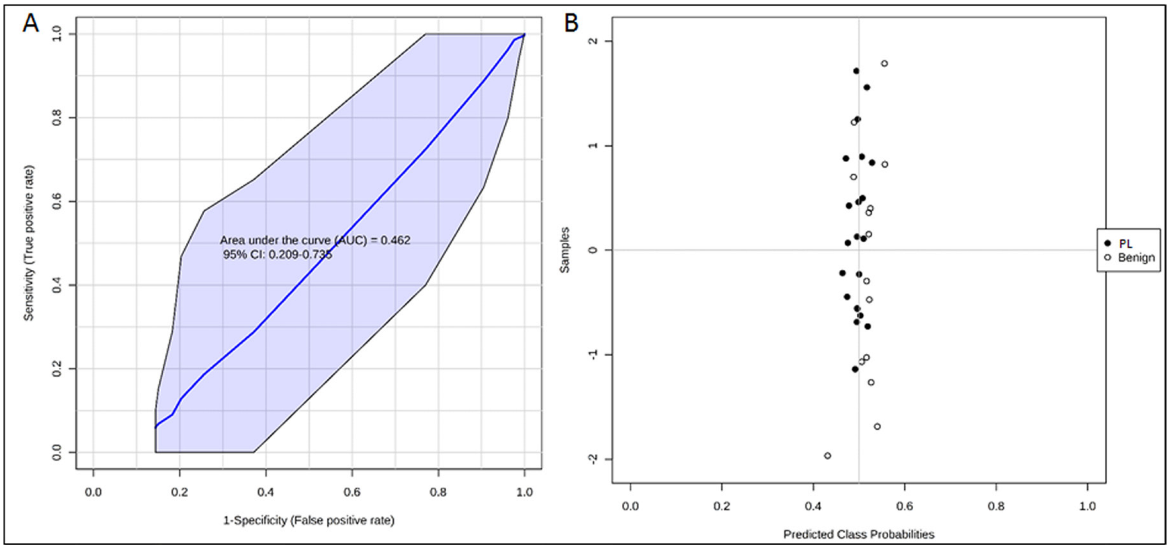

Supplementary Figure 5: ROC curve (**A**) and predicted class probabilities (**B**) between PL and Benign.

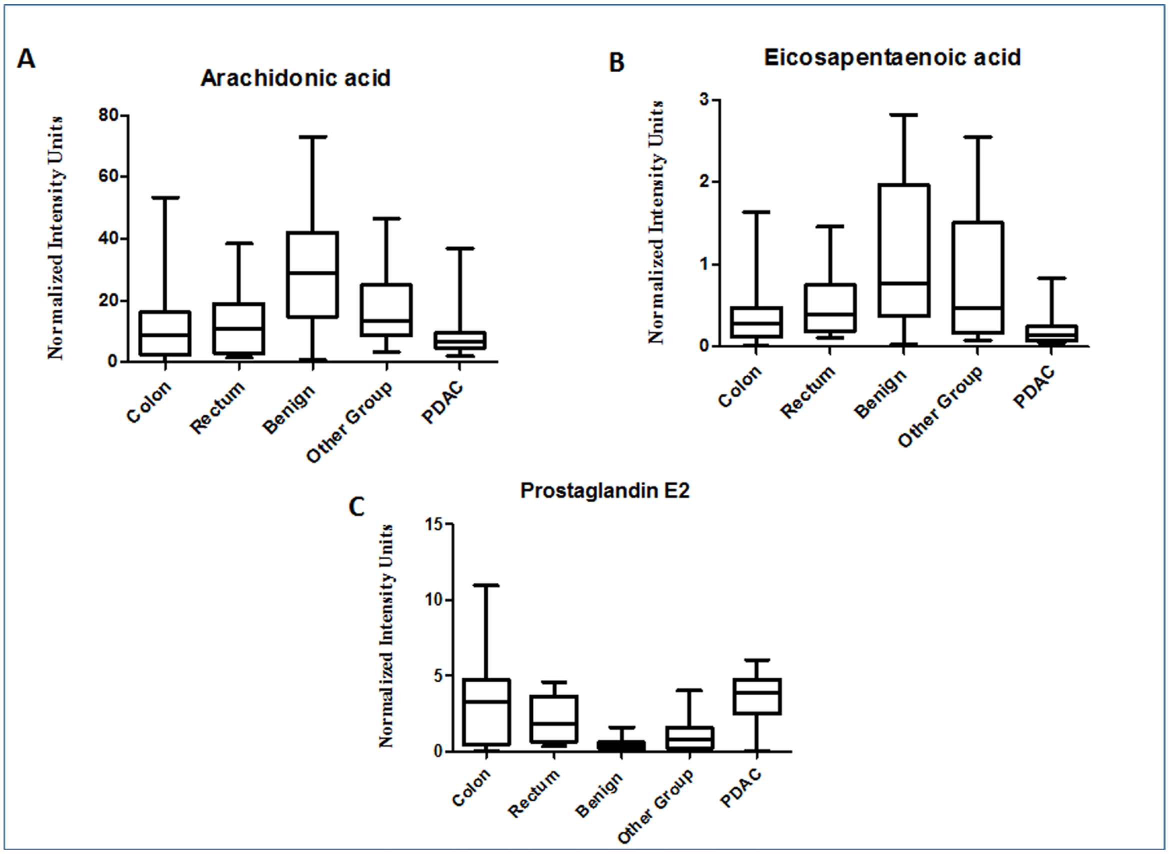

Supplementary Figure 6: Arachidonic acid pathway is dysregulated in pancreatic and colorectal cancer. Metabolites of the arachidonic acid pathway were quantified in tissue samples using multiple reaction monitoring mass spectrometry and visualized as box-and-whisker plots for all groups.

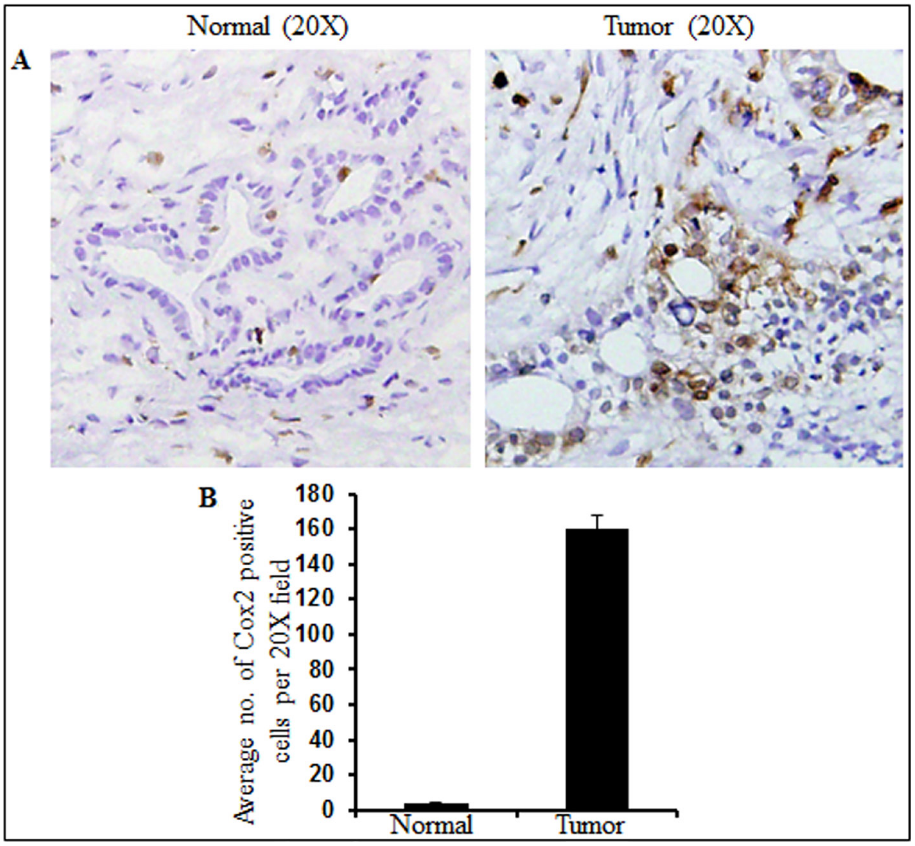

Supplementary Figure 7: Validation of dysregulation of arachidonic acid pathway in pancreatic cancer using immunohistochemistry. Pancreatic tumor tissue shows significantly more Cox2 staining relative to normal pancreatic tissue (p < 000000005). (**A**) IHC staining of pancreatic normal and tumor tissue. (**B**). Quantification.

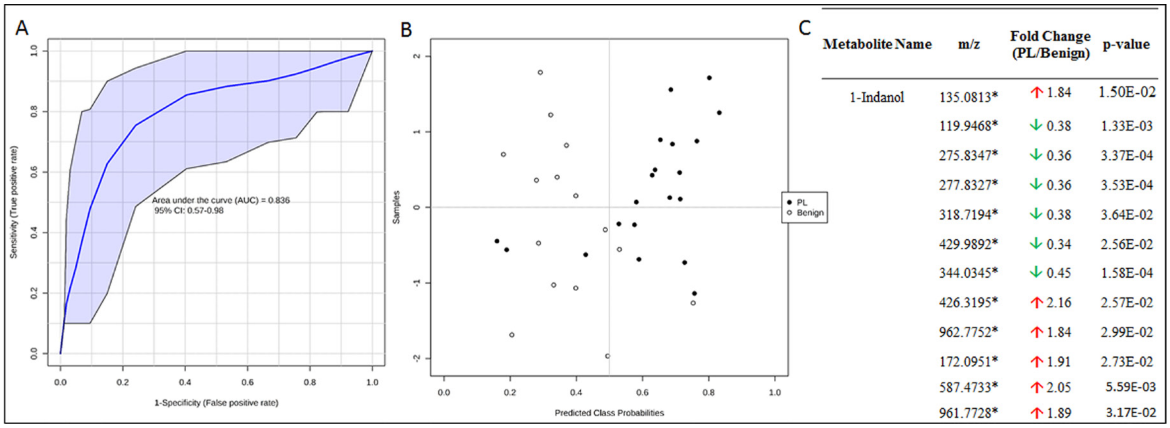

Metabolite Name	m/z	Fold Change (PL/Benign)	p-value
1-Indanol	135.0813*	↑ 1.84	1.50E-02
	119.9468*	↓ 0.38	1.33E-03
	275.8347*	↓ 0.36	3.37E-04
	277.8327*	↓ 0.36	3.53E-04
	318.7194*	↓ 0.38	3.64E-02
	429.9892*	↓ 0.34	2.56E-02
	344.0345*	↓ 0.45	1.58E-04
	426.3195*	↑ 2.16	2.57E-02
	962.7752*	↑ 1.84	2.99E-02
	172.0951*	↑ 1.91	2.73E-02
	587.4733*	↑ 2.05	5.59E-03
	961.7728*	↑ 1.89	3.17E-02

Supplementary Figure 8: ROC curve (**A**) and predicted class probabilities (**B**) between PL and Benign for a 12 metabolite panel (**C**) which were significant in PL compared to the benign pancreatic disease group.

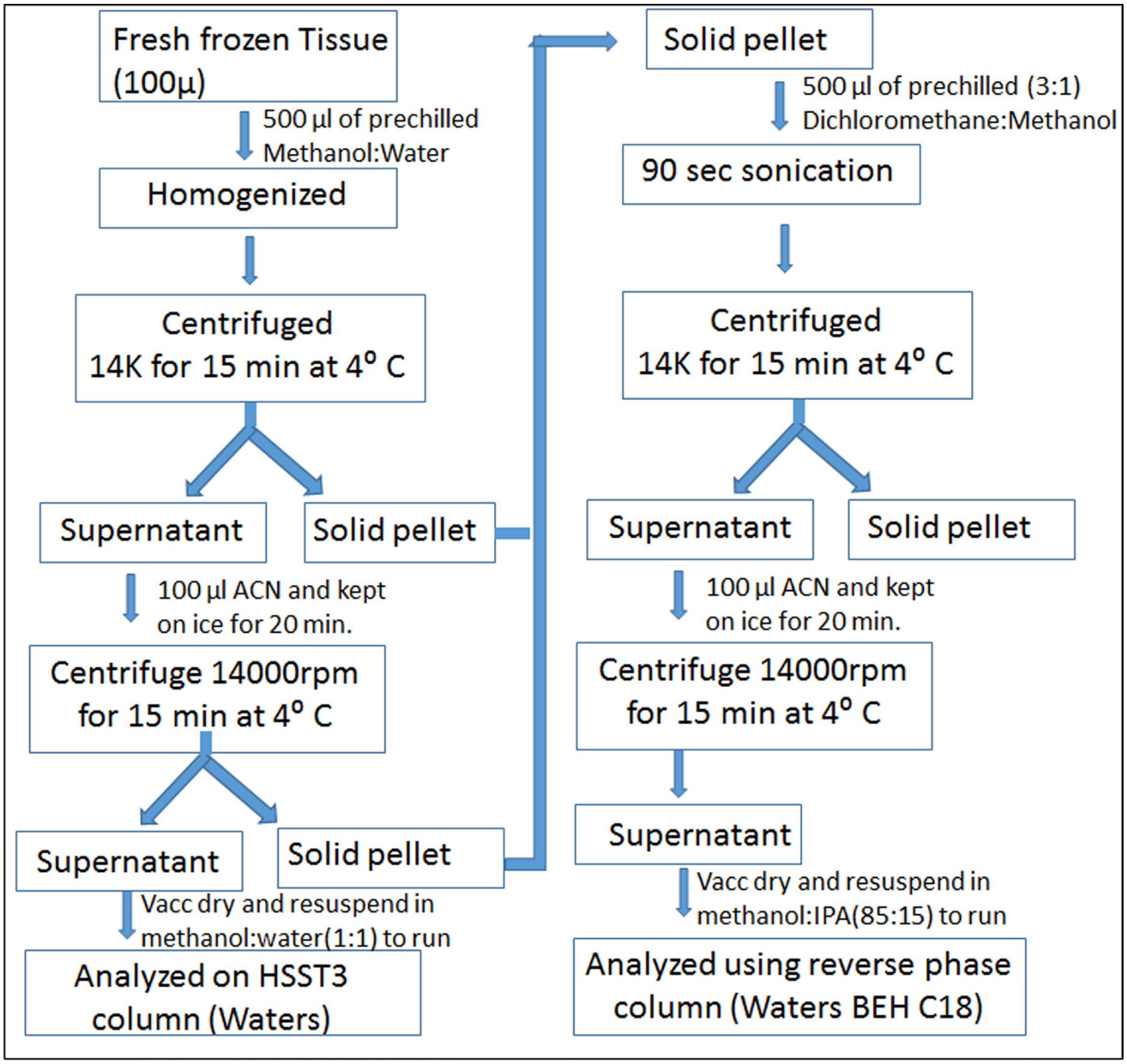

Supplementary Figure 9: Metabolite extraction schema.

Supplementary Table 1: Demographic and clinical information for each patient across all cohorts. See Supplementary_ Table_1

Supplementary Table 2: R^2 and Q^2 values for binary comparisons of benign versus disease in the organic extract in the positive ionization mode

Comparison	R^2	Q^2
Benign vs CRC	0.98	0.84
Benign vs PL	0.97	0.59
Benign vs PDAC	0.99	0.93

Supplementary Table 3: Metabolites found dysregulated in PDAC as compared to benign pancreatic conditions

		PDAC	
**Metabolite name**	**m/z**	**Fold change (PDAC/Benign)**	***p*-value**
PA(0-20:0/20:4)	737.5513	↑ 2.78	3.34E-09
Lactic Acid	89.0244	↑ 2.62	6.25E-09
17a-Hydroxypregnenolone	333.2391	↓ 0.23	7.39E-09
O-Arachidonoyl Glycidol	361.2726	↑ 3.06	3.56E-08
Glucose-6-Phosphate	259.0222	↑ 4.06	7.77E-08
PC(18:1/16:0)	760.585	↑ 2.35	4.40E-06
SM(d18:0/16:0)	705.5846	↑ 2.29	5.92E–06
PC(16:0/16:0)	734.5696	↑ 2.09	2.51E–05
PE(22:1/0:0)	536.3719	↓ 0.48	4.06E-05
5-hydroxytryptophan	221.0332	↓ 0.44	7.85E-05
PC(18:0/22:4)	838.6341	↑ 1.93	9.35E-05
SM(d18:1/16:0)	703.574	↑ 1.92	1.16E-04
LysoPE (0:0/18:2)	478.2947	↓ 0.24	1.27E-04
PC(P-20:0/19:1)	814.6697	↑ 2.01	2.76E-04
PE (16:0/0:0)	452.2776	↓ 0.28	2.80E-04
PE (17:0/0:0)	466.2952	↓ 0.33	0.0049
PC(P-20:0/19:0)	802.6702	↑ 1.81	0.0071
Palmitoyl-L-carnitine	400.341	↑ 2.04	0.019

Supplementary Table 4: Evaluation of the six metabolite biomarker panel in plasma samples using UPLC-MRM-MS

	PDAC		PL		CRC	
**Metabolite Name**	**Fold change (PDAC/Benign)**	***p*-value**	**Fold change (PL/Benign)**	***p*-value**	**Fold change (CRC/Benign)**	***p*-value**
PC(16:0/16:0)	1.59	0.55	0.74	0.07	0.92	0.82
PC(18:0/22:4)	0.88	0.23	0.79	0.11	0.73	0.01
SM(d18:1/16:0)	1.02	0.72	0.85	0.18	1.05	0.36

Supplementary Table 5: Metabolites found dysregulated in PDAC and CRC as compared to benign pancreatic conditions. See Supplementary_Table_5

Supplementary Table 6: Metabolites found dysregulated in PDAC, CRC, and PL as compared to benign pancreatic conditions

Metabolite name	m/z	PDAC		CRC		PL	
		Fold Change (PDAC/Benign)	*p*-value	Fold Change (CRC/Benign)	*p*-value	Fold Change (PL/Benign)	*p*-value
PE (P-16:0/20:4)	724.5261	↑ 4.75	1.86E-11	↓ 0.5	0.05	↓ 0.3	0.05
PA (0-20:0/20:4)	740.5252	↑ 5.18	1.52E-15	↑ 1.98	0.018	↑ 4.16	8.74E-05
PE(18:1/18:2)	742.5395	↑ 5.50	1.19E-13	↑ 2.10	0.029	↑ 6.33	6.26E-07
PE (18:0/18:1)	744.5545	↑ 5.80	1.91E-14	↑ 1.99	0.019	↑ 6.09	3.42E-07
PE (18:0/20:4)	766.5413	↑ 6.72	6.75E-14	↑ 1.87	0.012	↑ 5.32	2.86E-06
PS(0-20:0/17:1)	790.5891	↑ 4.12	4.61E-12	↑ 2.48	0.0204	↑ 3.42	2.18E-04
PC(16:0/22:6)	806.573	↑ 2.86	1.27E-07	↑ 1.89	0.0053	↑ 4.20	2.39E-04

Supplementary Table 7: Metabolites found dysregulated in PDAC and PL as compared to benign pancreatic conditions

Metabolite name	m/z	PDAC		PL	
		Fold change (PDAC/Benign)	*p*-value	Fold change (IPMN/Benign)	*p*-value
PC(18:0/20:4)	810.6026	↑ 3.85	3.08E–09	↑ 1.91	0.04
	167.9331	↓ 0.34	1.83E–05	↓ 0.30	9.06E–06
	181.9169	↓ 0.27	7.19E–09	↓ 0.29	1.17E–07
	184.9724	↓ 0.20	4.52E–09	↓ 0.28	2.69E–06
	301.8465	↓ 0.28	3.48E–07	↓ 0.28	1.11E–06
	311.8749	↓ 0.30	1.22E–06	↓ 0.26	3.27E–07
	313.8725	↓ 0.32	2.28E–06	↓ 0.27	6.40E–07
	381.0884	↓ 0.36	0.000382	↓ 0.42	0.00243
	322.8962	↓ 0.41	0.000234	↓ 0.45	0.000749
	531.3847	↑ 2.09	0.008638	↑ 2.48	0.008286
	547.3936	↓ 0.47	0.024242	↑ 1.86	0.047842
	571.8569	↑ 1.94	0.000132	↑ 1.84	0.000694
	690.5951	↑ 2.50	4.01E–06	↑ 1.86	0.023543
	741.8297	↑ 1.84	0.000295	↑ 1.83	0.000495
	826.8038	↑ 1.96	0.000182	↑ 1.90	0.000568
	457.3215	↓ 0.37	0.001125	↓ 0.42	0.002855

Supplementary Table 8: Metabolites found dysregulated in PL as compared to benign pancreatic conditions

		PL	
**Metabolite name**	**m/z**	**Fold change (PL/Benign)**	***p*-value**
1-Indanol	135.0813	↑ 1.84	1.50E–02
	119.9468	↓ 0.38	1.33E–03
	139.919	↓ 0.49	6.83E–03
	157.907	↓ 0.38	3.05E–04
	165.9238	↓ 0.45	3.77E–04
	178.8344	↓ 0.43	2.20E–03
	246.9501	↓ 0.44	2.25E–02
	275.8347	↓ 0.36	3.37E–04
	277.8327	↓ 0.36	3.53E–04
	281.9119	↓ 0.38	4.43E–04
	287.8619	↓ 0.41	1.89E–04
	295.8924	↓ 0.44	5.36E–04
	297.8897	↓ 0.39	1.99E–04
	318.7194	↓ 0.38	3.64E–02
	429.9892	↓ 0.34	2.56E–02
	433.2461	↓ 0.50	2.42E–02
	512.0777	↓ 0.17	4.72E–02
	548.0581	↓ 0.44	9.46E–03
	573.0373	↓ 0.44	3.59E–03
	344.0345	↓ 0.45	1.58E–04
	426.3195	↑ 2.16	2.57E–02
	962.7752	↑ 1.84	2.99E–02
	172.0951	↑ 1.91	2.73E–02
	587.4733	↑ 2.05	5.59E–03
	961.7728	↑ 1.89	3.17E–02

Supplementary Table 9: All metabolites that were found to be dysregulated across any of the 3 disease groups (Pancreatic Ductal Adenocarcinoma, Pancreatic Lesions, Colorectal Cancer) as compared to the benign pancreatic condition. See Supplementary_Table_9
