# Supplementary material for: Metabolomics based predictive classifier for early detection of pancreatic ductal adenocarcinoma
Source: Oncotarget. 2018 May 1;9(33):23078–90. doi: 10.18632/oncotarget.25212 (PMC5955422; doi:10.18632/oncotarget.25212)
Supplement: Supplementary file 2 [file oncotarget-09-23078-s002.docx]

**Supplementary Table 1**: Demographic and clinical information for each patient across all cohorts.

| Status | Case ID | Block ID | Tissue | Diagnosis | Sample | Age | Sex | DM | Jaundice | Chemo | Radiation | Tumor % | Normal % | Necrosis % | Other % | What is Other? | Histology* | Diagnosis* |
| --- | --- | --- | --- | --- | --- | --- | --- | --- | --- | --- | --- | --- | --- | --- | --- | --- | --- | --- |
| Benign | 1A057 | Tn310 | Pancreas | Normal | Normal | 40 | M | DM2 | no | n/a | n/a | 0 | 100 | 0 | 0 |  | 3 | 3 |
| Benign | 1A363 | Tn310 | Pancreas | Pancreatitis | Other | 18 | F | DM1 | no | n/a | n/a | 0 | 0 | 0 | 100 | Pancreatitis | 3 | 3 |
| Benign | 1A253 | Tn310 | Pancreas | Normal | Normal | 66 | F | no | yes | n/a | n/a | 0 | 100 | 0 | 0 |  | 3 | 3 |
| Benign | 1A266 | Tn307 | Pancreas | Normal | Normal | 25 | F | no | no | n/a | n/a | 0 | 100 | 0 | 0 |  | 3 | 3 |
| Benign | 1A385 | Tn309 | Pancreas | Pancreatitis | Other | 50 | F | no | no | n/a | n/a | 0 | 50 | 0 | 50 | Pancreatitis | 3 | 3 |
| Benign | 1A356 | Tn308 | Pancreas | Normal | Normal | 56 | F | no | no | n/a | n/a | 0 | 100 | 0 | 0 |  | 3 | 3 |
| Benign | 1A388 | Tn9 | Pancreas | Normal | Normal | 57 | F | no | no | n/a | n/a | 0 | 100 | 0 | 0 |  | 3 | 3 |
| Benign | 1A395 | Tn309 | Pancreas | Pancreatitis | Other | 56 | M | no | no | n/a | n/a | 0 | 0 | 0 | 100 | Pancreatitis | 3 | 3 |
| Benign | 1A401 | Tn308 | Pancreas | Normal | Normal | 40 | F | no | no | n/a | n/a | 0 | 100 | 0 | 0 |  | 3 | 3 |
| Benign | 1A414 | Tn10 | Pancreas | Normal | Normal | 79 | M | no | yes | n/a | n/a | 0 | 100 | 0 | 0 |  | 3 | 3 |
| Benign | 1A506 | Tn310 | Pancreas | Pancreatitis | Other | 48 | M | DM1 | no | n/a | n/a | 0 | 0 | 0 | 100 | Pancreatitis | 3 | 3 |
| Benign | 1A462 | Tn308 | Pancreas | Normal | Normal | 41 | F | no | no | n/a | n/a | 0 | 90 | 0 | 10 |  | 3 | 3 |
| Benign | 1A602 | Tn307 | Pancreas | Normal | Normal | 52 | M | DM2 | no | n/a | n/a | 0 | 100 | 0 | 0 |  | 3 | 3 |
| Benign | 1A743 | Tn10 | Pancreas | Pancreatitis | Other | 60 | F | no | no | n/a | n/a | 0 | 0 | 0 | 100 | Pancreatitis | 3 | 3 |
| Benign | 1A273 | Tp2 | Pancreas | Serous Cystic Neoplasm | Tumor & Normal | 73 | M | DM2 | no | n/a | n/a | 60 | 40 | 0 | 0 |  | 3 | 3 |
| CRC | 1A103 | Tp3 | Colon | Adenocarcinoma | Tumor & Normal | 64 | M | no | no | no | no | 70 | 30 | 30 | 0 |  | 1 | 1 |
| CRC | 1A107 | Tp303 | Colon | Adenocarcinoma | Tumor Only | 41 | M | no | no | no | no | 100 | 0 | 10 | 0 |  | 1 | 1 |
| CRC | 1A192 | Tp404 | Colon | Adenocarcinoma | Tumor & Normal | 36 | F | no | no | no | no | 90 | 10 | 0 | 0 |  | 1 | 1 |
| CRC | 1A115 | Tp1 | Rectum | Carcinoma | Tumor Only | 47 | F | no | no | no | no | 100 | 0 | 5 | 0 |  | 1 | 1 |
| CRC | 1A153 | Tc5 | Rectum | Carcinoma | Tumor Only | 77 | M | DM2 | no | no | no | 100 | 0 | 0 | 0 |  | 1 | 1 |
| CRC | 1A227 | Tp502 | Colon | Adenocarcinoma | Tumor | 60 | M | no | no | no | no | 80 | 0 | 5 | 20 | Granulation Tissue | 1 | 1 |
| CRC | 1A267 | Tp301 | Colon | Adenocarcinoma | Tumor & Normal | 76 | F | no | no | no | no | 80 | 20 | 40 | 0 |  | 1 | 1 |
| CRC | 1A298 | Tp303 | Colon | Adenocarcinoma | Tumor Only | 51 | F | no | no | no | no | 100 | 0 | 0 | 0 |  | 1 | 1 |
| CRC | 1A187 | Tp302 | Rectum | Carcinoma | Tumor | 60 | F | no | no | yes | no | 90 | 0 | 0 | 10 | Stroma | 1 | 1 |
| CRC | 1A246 | Tp2 | Rectum | Carcinoma | Tumor Only | 82 | M | DM2 | no | no | no | 100 | 0 | 5 | 0 |  | 1 | 1 |
| CRC | 1A304 | Tp303 | Colon | Adenocarcinoma | Tumor Only | 28 | M | no | no | no | no | 100 | 0 | 0 | 0 |  | 1 | 1 |
| CRC | 1A335 | Tp302 | Colon | Adenocarcinoma | Tumor & Normal | 85 | M | no | no | no | no | 80 | 0 | 10 | 20 | Inflamatory Stromal Tissue | 1 | 1 |
| CRC | 1A295 | Tp2 | Rectum | Carcinoma | Tumor & Normal | 52 | M | no | no | no | no | 80 | 20 | 0 | 0 |  | 1 | 1 |
| CRC | 1A337 | Tp2 | Rectum | Carcinoma | Tumor Only | 66 | F | no | no | no | no | 100 | 0 | 0 | 0 |  | 1 | 1 |
| CRC | 1A408 | Tp1 | Colon | Adenocarcinoma | Tumor Only | 68 | F | no | no | no | no | 100 | 0 | 0 | 0 |  | 1 | 1 |
| CRC | 1A411 | Tp302 | Colon | Adenocarcinoma | Tumor & Normal | 80 | F | no | no | no | no | 95 | 5 | 5 | 0 |  | 1 | 1 |
| CRC | 1A425 | Tp2 | Colon | Adenocarcinoma | Tumor & Normal | 61 | F | no | no | no | no | 95 | 5 | 5 | 0 |  | 1 | 1 |
| CRC | 1A347 | Tp1 | Rectum | Carcinoma | Tumor & Normal | 36 | F | no | no | yes | yes | 50 | 20 | 0 | 30 | Stroma | 1 | 1 |
| CRC | 1A512 | Tp401 | Rectum | Carcinoma | Tumor | 78 | F | no | no | no | no | 90 | 0 | 2 | 10 | Stroma | 1 | 1 |
| CRC | 1A438 | Tc5 | Colon | Adenocarcinoma | Tumor Only | 62 | F | no | no | no | no | 100 | 0 | 10 | 0 |  | 1 | 1 |
| CRC | 1A453 | Tp2 | Colon | Adenocarcinoma | Tumor Only | 61 | M | no | no | no | no | 100 | 0 | 5 | 0 |  | 1 | 1 |
| CRC | 1A456 | Tp602 | Colon | Adenocarcinoma | Tumor Only | 70 | F | no | no | yes | no | 100 | 0 | 40 | 0 |  | 1 | 1 |
| CRC | 1A603 | Tp301 | Colon | Adenocarcinoma | Tumor | 44 | F | no | no | no | no | 80 | 0 | 0 | 20 | Stroma | 1 | 1 |
| CRC | 1A615 | Tp302 | Colon | Adenocarcinoma | Tumor & Normal | 31 | M | no | no | no | no | 80 | 10 | 5 | 10 | Stroma | 1 | 1 |
| CRC | 1A630 | Tp3 | Colon | Adenocarcinoma | Tumor | 65 | F | no | no | no | no | 50 | 0 | 20 | 50 | Stroma | 1 | 1 |
| CRC | 1A477 | Tc405 | Colon | Adenocarcinoma | Tumor Only | 85 | F | no | no | no | no | 100 | 0 | 5 | 0 |  | 1 | 1 |
| CRC | 1A495 | Tc5 | Colon | Adenocarcinoma | Tumor | 58 | M | no | no | no | no | 70 | 0 | 5 | 30 | Smooth Muscle | 1 | 1 |
| CRC | 1A548 | Tp304 | Colon | Adenocarcinoma | Tumor | 56 | F | no | no | no | no | 80 | 0 | 0 | 20 | Stroma | 1 | 1 |
| PL | 1A007 | Tc5 | Pancreas | IPMN | Tumor & Normal | 61 | M | no | no | n/a | n/a | 5 | 2 | 0 | 40 | Lymphoid component | 4 | 4 |
| PL | 1A037 | Tc5 | Pancreas | IPMN | Tumor | 72 | F | DM2 | no | n/a | n/a | 0 | 100 | 0 | 0 |  | 3 | 4 |
| PL | 1A034 | Tp4 | Pancreas | Mucinous Cystic Neoplasm | Tumor | 53 | F | no | no | n/a | n/a | 0 | 100 | 0 | 0 |  | 3 | 4 |
| PL | 1A039 | Tc5 | Pancreas | IPMN | Tumor | 73 | F | no | no | n/a | n/a | 30 | 0 | 0 | 70 | Fibrosis | 4 | 4 |
| PL | 1A040 | Tc5 | Pancreas | IPMN | Tumor & Normal | 78 | F | no | no | n/a | n/a | 60 | 40 | 0 | 0 |  | 4 | 4 |
| PL | 1A081 | Tp1 | Pancreas | Mucinous Cystic Neoplasm | Tumor | 48 | F | no | no | n/a | n/a | 0 | 100 | 0 | 0 |  | 3 | 4 |
| PL | 1A047 | Tp2 | Pancreas | IPMN | Tumor | 80 | F | no | no | n/a | n/a | 0 | 95 | 0 | 5 |  | 3 | 4 |
| PL | 1A050 | Tn10 | Pancreas | IPMN | Tumor | 60 | F | no | no | n/a | n/a | 0 | 100 | 0 | 0 |  | 3 | 4 |
| PL | 1A596 | Tp2 | Pancreas | Mucinous Cystic Neoplasm | Tumor & Normal | 24 | F | no | no | n/a | n/a | 5 | 95 | 0 | 0 |  | 4 | 4 |
| PL | 1A381 | Tp302 | Pancreas | IPMN | Tumor | 63 | M | no | no | n/a | n/a | 70 | 0 | 0 | 30 | Fibrosis | 4 | 4 |
| PL | 1A403 | Tp2 | Pancreas | IPMN | Tumor | 47 | M | no | no | n/a | n/a | 70 | 0 | 0 | 30 | Fibrosis, inflammation | 4 | 4 |
| PL | 1A439 | Tn310 | Pancreas | IPMN | Tumor | 50 | F | no | no | n/a | n/a | 0 | 100 | 0 | 0 |  | 3 | 4 |
| PL | 1A465 | Tp2 | Pancreas | IPMN | Tumor | 70 | M | no | no | n/a | n/a | 20 | 0 | 0 | 80 | Fibrosis | 4 | 4 |
| PL | 1A524 | Tn8 | Pancreas | IPMN | Tumor & Normal | 58 | M | no | no | n/a | n/a | 15 | 75 | 0 | 10 | Fibrosis | 4 | 4 |
| PL | 1A525 | Tn308 | Pancreas | IPMN | Tumor | 45 | F | no | no | n/a | n/a | 0 | 100 | 0 | 0 |  | 3 | 4 |
| PL | 1A440 | Tp2 | Pancreas | Pseudopapillary Neoplasm | Tumor Only | 36 | M | no | no | n/a | n/a | 100 | 0 | 0 | 0 |  | 4 | 4 |
| PL | 1A119 | Tc305 | Pancreas | Cystic Papillary Neoplasm | Tumor Only | 50 | F | no | no | n/a | n/a | 100 | 0 | 0 | 0 |  | 4 | 4 |
| PL | 1A319 | Tp3 | Pancreas | Cystic Papillary Neoplasm | Tumor & Normal | 50 | F | no | no | n/a | n/a | 5 | 5 | 0 | 90 | Fibrosis | 4 | 4 |
| PL | 1A697 | Tp2 | Pancreas | IPMN | Tumor & Normal | 71 | M | no | no | n/a | n/a | 20 | 30 | 0 | 50 | Fibrosis | 4 | 4 |
| PL | 1A702 | Tn309 | Pancreas | IPMN | Tumor | 69 | F | no | no | n/a | n/a | 0 | 100 | 0 | 0 |  | 3 | 4 |
| PDAC | 1A043 | Tp4 | Pancreas | Adenocarcinoma | Tumor | 69 | M | DM2 | no | no | no | 60 | 0 | 0 | 40 | Fibrosis | 2 | 2 |
| PDAC | 1A120 | Tp3 | Pancreas | Adenocarcinoma | Tumor | 66 | M | no | no | no | no | 60 | 0 | 0 | 40 | Fibrosis | 2 | 2 |
| PDAC | 1A195 | Tp303 | Pancreas | Adenocarcinoma | Tumor | 56 | M | no | yes | no | no | 50 | 0 | 0 | 50 | Chronic fibrosis | 2 | 2 |
| PDAC | 1A199 | Tc305 | Pancreas | Adenocarcinoma | Tumor & Normal | 73 | M | no | no | no | no | 80 | 15 | 0 | 5 | Chronic inflammation, fibrosis | 2 | 2 |
| PDAC | 1A225 | Tp403 | Pancreas | Adenocarcinoma | Tumor | 79 | M | no | no | no | no | 50 | 0 | 50 | 50 | Chronic inflammation, fibrosis | 2 | 2 |
| PDAC | 1A277 | Tc305 | Pancreas | Adenocarcinoma | Tumor & Normal | 54 | F | DM2 | yes | no | no | 80 | 10 | 0 | 10 | Chronic inflammation, fibrosis | 2 | 2 |
| PDAC | 1A290 | Tc305 | Pancreas | Adenocarcinoma | Tumor & Normal | 65 | M | no | yes | no | no | 85 | 5 | 0 | 10 | Chronic inflammation, fibrosis | 2 | 2 |
| PDAC | 1A293 | Tc6 | Pancreas | Adenocarcinoma | Tumor & Normal | 54 | M | no | yes | no | no | 85 | 5 | 0 | 10 | Chronic inflammation, fibrosis | 2 | 2 |
| PDAC | 1A302 | Tc306 | Pancreas | Adenocarcinoma | Tumor & Normal | 49 | M | no | no | no | no | 70 | 10 | 0 | 20 | Chronic inflammation, fibrosis | 2 | 2 |
| PDAC | 1A321 | Tc305 | Pancreas | Adenocarcinoma | Tumor & Normal | 59 | F | no | yes | no | no | 70 | 5 | 0 | 25 | Chronic inflammation, fibrosis | 2 | 2 |
| PDAC | 1A330 | Tp302 | Pancreas | Adenocarcinoma | Tumor & Normal | 43 | F | DM2 | no | no | no | 60 | 10 | 0 | 30 | Chronic inflammation, fibrosis | 2 | 2 |
| PDAC | 1A333 | Tp3 | Pancreas | Adenocarcinoma | Tumor & Normal | 65 | M | no | no | no | no | 75 | 5 | 20 | 20 | Chronic inflammation, fibrosis | 2 | 2 |
| PDAC | 1A357 | Tp302 | Pancreas | Adenocarcinoma | Tumor & Normal | 46 | M | DM2 | yes | no | no | 80 | 10 | 50 | 10 | Chronic inflammation, fibrosis | 2 | 2 |
| PDAC | 1A375 | Tc306 | Pancreas | Adenocarcinoma | Tumor Only | 82 | M | no | yes | no | no | 100 | 0 | 10 | 0 |  | 2 | 2 |
| PDAC | 1A431 | Tp401 | Pancreas | Adenocarcinoma | Tumor & Normal | 54 | M | DM2 | no | no | no | 75 | 10 | 0 | 15 | Chronic inflammation, fibrosis | 2 | 2 |
| PDAC | 1A447 | Tp301 | Pancreas | Adenocarcinoma | Tumor | 62 | F | no | no | no | no | 60 | 0 | 20 | 40 | Fibrosis | 2 | 2 |
| PDAC | 1A475 | Tp1 | Pancreas | Adenocarcinoma | Tumor | 66 | F | no | no | no | no | 90 | 0 | 50 | 10 | Chronic inflammation, fibrosis | 2 | 2 |
| PDAC | 1A551 | Tp2 | Pancreas | Adenocarcinoma | Tumor | 66 | M | no | yes | no | no | 50 | 0 | 0 | 50 | Chronic inflammation, fibrosis | 2 | 2 |
| PDAC | 1A569 | Tp301 | Pancreas | Adenocarcinoma | Tumor | 62 | F | DM1 | yes | no | no | 50 | 0 | 10 | 50 | Fibrosis | 2 | 2 |
| * 1 = Colorectal cancer, 2 = Pancreatic Ductal Adenocarcinoma, 3 = Benign Pancreaic Condition, 4 = Pancreatic Lesion. | | | | | | | | | | | | | | | | | | |
