# Supplementary material for: Metabolomics based predictive classifier for early detection of pancreatic ductal adenocarcinoma
Source: Oncotarget. 2018 May 1;9(33):23078–90. doi: 10.18632/oncotarget.25212 (PMC5955422; doi:10.18632/oncotarget.25212)
Supplement: Supplementary file 3 [file oncotarget-09-23078-s003.docx]

**Supplementary Table 5:** Metabolites found dysregulated in PDAC and CRC as compared to benign pancreatic conditions.

|  | | **PDAC** | | **CRC** | |
| --- | --- | --- | --- | --- | --- |
| **Metabolite Name** | **m/z** | **Fold Change (PDAC/Benign)** | **p-value** | **Fold Change (CRC/Benign)** | **p-value** |
| Linoleic Acid | 279.232 | 🡫 0.17 | 1.24E-09 | 🡫 0.30 | 1.23E-05 |
| Arachidonic Acid | 303.232 | 🡫 0.33 | 3.91E-04 | 🡫 0.45 | 0.0054 |
| Glucosamine | 214.05 | 🡫 0.32 | 0.0017 | 🡫 0.44 | 0.0132 |
| Cytidine | 242.08 | 🡫 0.32 | 4.07E-04 | 🡫 0.40 | 1.27E-03 |
| LysoPE (0:0/18:0) | 482.324 | 🡫 0.23 | 1.59E-05 | 🡫 0.34 | 4.33E-04 |
| Lyso PC (16:1) | 494.324 | 🡫 0.14 | 9.64E-07 | 🡫 0.28 | 3.87E-04 |
| LysoPC(18:2) | 520.34 | 🡫 0.015 | 3.04E-06 | 🡫 0.24 | 8.44E-05 |
| PC(20:5/0:0) | 542.322 | 🡫 0.20 | 2.33E-05 | 🡫 0.26 | 1.02E-04 |
| Lyso PC (17:0) | 510.355 | 🡫 0.09 | 7.74E-08 | 🡫 0.12 | 2.24E-07 |
| PE (16:0/18:1) | 716.527 | 🡩 4.24 | 8.34E-12 | 🡩 4.49 | 3.63E-05 |
| Oleamide | 282.278 | 🡩 4.03 | 1.01E-07 | 🡩 3.21 | 0.0083 |
| PC(18:1/18:1) | 786.601 | 🡩 2.79 | 8.61E-07 | 🡩 2.08 | 0.0112 |
| PC(18:0/18:1) | 788.617 | 🡩 3.74 | 1.15E-09 | 🡩 2.22 | 0.0138 |
| Taurine | 124.007 | 🡩 2.37 | 6.61E-08 | 🡩 2.11 | 5.00E-05 |
| PE (P-16:0/0:0) | 436.283 | 🡫 0.21 | 1.09E-05 | 🡫 0.32 | 5.89E-04 |
| PE (P-18:0/0:0) | 464.314 | 🡫 0.24 | 5.28E-05 | 🡫 0.39 | 0.0043 |
| PE (20:0/0:0) | 508.34 | 🡫 0.21 | 4.73E-05 | 🡫 0.46 | 0.028 |
| PI (16:0/0:0) | 571.288 | 🡫 0.07 | 1.79E-09 | 🡫 0.12 | 1.13E-07 |
| LysoPC(16:0) | 496.339 | 🡫 0.30 | 0.0051 | 🡫 0.17 | 1.57E-06 |
| CPA(18:0) | 421.273 | 🡫 0.20 | 6.69E-10 | 🡫 0.34 | 1.04E-05 |
| Glycerophospho-N-Palmitoyl Ethanolamine | 454.293 | 🡫 0.19 | 7.78E-07 | 🡫 0.40 | 0.0013 |
| PE (18:1/0:0) | 480.311 | 🡫 0.21 | 2.63E-05 | 🡫 0.48 | 0.0287 |
| LysoPE (0:0/20:3) | 504.306 | 🡫 0.29 | 9.93E-06 | 🡫 0.44 | 0.0017 |
| PC(18:3) | 518.319 | 🡫 0.09 | 3.78E-09 | 🡫 0.20 | 6.53E-06 |
| Lyso PC(0:0/18:1) | 522.355 | 🡫 0.13 | 1.62E-06 | 🡫 0.23 | 8.39E-05 |
| LysoPC(0:0/18:0) | 524.371 | 🡫 0.12 | 1.44E-08 | 🡫 0.14 | 3.46E-08 |
| LysoPC(20:4) | 544.34 | 🡫 0.12 | 4.02E-07 | 🡫 0.25 | 1.68E-04 |
| LysoPC(20:1/0:0) | 550.386 | 🡫 0.12 | 8.92E-07 | 🡫 0.25 | 2.13E-05 |
| PS(22:1/0:0) | 578.345 | 🡫 0.44 | 0.0044 | 🡫 0.20 | 8.42E-09 |
| PI(18:1/0:0) | 597.304 | 🡫 0.32 | 0.0096 | 🡫 0.32 | 0.0025 |
| PI(18:0/0:0) | 599.32 | 🡫 0.44 | 0.04957 | 🡫 0.16 | 9.29E-08 |
| LysoPC(20:0/0:0) | 552.402 | 🡫 0.21 | 1.22E-07 | 🡫 0.37 | 9.83E-05 |
| PS(20:1/0:0) | 550.313 | 🡫 0.49 | 0.02279 | 🡫 0.24 | 3.75E-07 |
| PC(P-16:0/15:1) | 702.546 | 🡩 4.19 | 8.48E-11 | 🡩 3.40 | 9.02E-04 |
| PC(P-16:0/17:2) | 728.56 | 🡩 2.65 | 3.25E-06 | 🡩 4.03 | 8.18E-04 |
| PC(16:1/16:0) | 732.559 | 🡩 1.95 | 1.06E-04 | 🡩 2.19 | 0.0438 |
| PC(16:0/18:0) | 762.595 | 🡩 2.86 | 5.94E-08 | 🡩 2.02 | 0.0332 |
| PC(14:1/22:2) | 784.585 | 🡩 2.60 | 8.19E-07 | 🡩 2.12 | 0.0073 |
| PS(18:1/18:0) | 790.559 | 🡩 2.24 | 5.97E-05 | 🡩 4.45 | 0.0376 |
| PC(18:1/20:4) | 808.586 | 🡩 3.69 | 1.87E-09 | 🡩 2.10 | 0.011 |
| PC(22:0/O-18:1) | 828.689 | 🡩 2.18 | 0.0023 | 🡩 3.73 | 1.35E-04 |
| PC(P-18:0/22:0) | 830.697 | 🡩 2.18 | 2.33E-05 | 🡩 3.78 | 3.25E-04 |
| LysoPE(20:4/0:0) | 500.278 | 🡩 5.23 | 4.97E-13 | 🡩 9.93 | 2.06E-04 |
| LysoPE(22:4/0:0) | 528.309 | 🡩 4.49 | 6.67E-11 | 🡩 2.46 | 0.028 |
| PE (P-16:0/18:1) | 700.528 | 🡩 5.12 | 1.09E-11 | 🡩 4.24 | 4.26E-05 |
| PE (P-16:0/20:4) | 722.513 | 🡩 5.76 | 3.43E-11 | 🡩 3.33 | 1.52E-04 |
| PE (O-16:0/20:4) | 726.542 | 🡩 3.94 | 1.25E-11 | 🡩 4.98 | 4.12E-05 |
| PE (P-18:0/20:4) | 750.545 | 🡩 4.47 | 1.27E-11 | 🡩 3.05 | 0.0017 |
| PE (P-18:0/22:6) | 774.541 | 🡩 3.96 | 3.21E-11 | 🡩 3.36 | 0.0015 |
| Arachidyl carnitine | 456.401 | 🡩 4.97 | 2.59E-06 | 🡩 3.08 | 0.038 |
