# Supplementary material for: Metabolomics based predictive classifier for early detection of pancreatic ductal adenocarcinoma
Source: Oncotarget. 2018 May 1;9(33):23078–90. doi: 10.18632/oncotarget.25212 (PMC5955422; doi:10.18632/oncotarget.25212)
Supplement: Supplementary file 4 [file oncotarget-09-23078-s004.docx]

**Supplementary Table 9**: All metabolites that were found to be dysregulated across any of the 3 disease groups (Pancreatic Ductal Adenocarcinoma, Pancreatic Lesions, Colorectal Cancer) as compared to the benign pancreatic condition.

| **Metabolite** | **m/z** | **Mass Error (ppm)** | **Retention Time (min)** | **Phase** | **Mode** | **CID Fragments** |
| --- | --- | --- | --- | --- | --- | --- |
| Lactic Acid | 89.0244 | 0 | 0.78 | Aqueous | Neg | 87.0090, 71.0143 |
| Taurine | 124.0071 | 2 | 0.78 | Aqueous | Neg | 106.9814, 79.9570 |
| 1-Indanol | 135.0813 | 6 | 0.92 | Aqueous | Pos | 135.0800, 105.0700, 91.0542, 49.0073, 39.0229 |
| Glucosamine | 214.0497 | 4 | 0.77 | Aqueous | Neg | 96.9608 |
| 5-Hydroxy-L-Tryptophan | 221.0932 | 5 | 0.88 | Aqueous | Pos | 133.0540,104.0550,102.0460,89.0380,79.0510 |
| Cytidine | 242.0798 | 6 | 0.77 | Aqueous | Neg | 152.0460, 110.0370 |
| Glucose-6-Phosphate | 259.0222 | 0 | 0.94 | Aqueous | Neg | 222.9989, 198.9984, 96.9689, 78.9587 |
| Linoleic Acid | 279.2322 | 2 | 8.12 | Organic | Neg | 279.2330, 261.1 |
| Oleamide | 282.278 | 4 | 6.39 | Organic | Pos | 282.2787, 281.1781, 121.1027, 111.1180, 95.0859, 67.0538 |
| Omega-3 Arachidonic Acid | 303.2323 | 2 | 8.02 | Organic | Neg | 303.2359, 259.2420 |
| 17a-Hydroxypregnenolone | 333.2391 | 9 | 7.93 | Organic | Pos | 333.2414, 332.2314, 119.0871, 111.0861, 109.0979, 107.0846, 105.0694, 95.0856, 93.0693, 91.0542, 79.0538, 67.0545 |
| O-Arachidonoyl Glycidol | 361.2726 | 3 | 6.21 | Organic | Pos | 361.2732, 269.2261, 203.1785, 133.1006, 119.0852, 107.0854, 105.0707, 95.0856, 93.0700, 91.0544,81.0701, 79.0545, 67.0546 |
| Palmitoyl-L-carnitine | 400.341 | 2 | 7.33 | Organic | Pos | 400.3416, 341.2674, 85.0280, 60.0806 |
| CPA(18:0) | 421.2731 | 4 | 7.38 | Organic | Pos | 421.2710, 267.2680, 155.0100, 85.1012 |
| PE(P-16:0/0:0) | 436.2826 | 1 | 6.84 | Organic | Neg | 436.2834, 196.0380, 152.9958, 140.0118, 122.0013, 78.9591 |
| PE(16:0/0:0) | 452.2776 | 1 | 6.61 | Organic | Neg | 452.2783, 255.2330, 214.0486, 196.0380, 152.9958, 140.0118, 122.0013, 78.9591 |
| Glycerophospho-N-Palmitoyl Ethanolamine | 454.2931 | 0 | 6.61 | Organic | Pos | 454.2932, 282.2792, 109.1022, 95.0890, 71.0855 |
| Arachidyl carnitine | 456.4008 | 8 | 9.16 | Organic | Pos | 456.4050, 144.1020, 85.0284 |
| PE(P-18:0/0:0) | 464.3138 | 1 | 7.66 | Organic | Neg | 464.3147, 196.0380, 152.9958, 140.0118, 122.0013, 78.9591 |
| PE(17:0) | 466.2952 | 2 | 6.97 | Organic | Neg | 466.2939, 2669.4786, 214.0486, 196.0380, 140.0118, 122.0013, 78.9591 |
| LysoPE(18:2) | 478.2947 | 3 | 6.3 | Organic | Pos | 478.2930, 460.2820, 337.2740, 198.0530 |
| PE(18:1) | 480.3108 | 4 | 6.8 | Organic | Pos | 480.3069, 462.2961, 419.2559, 339.2885, 308.2942, 265.2506, 247.2439, 216.0625 |
| LysoPE(18:0) | 482.3242 | 0 | 7.42 | Organic | Pos | 482.3240, 464.3140, 421.2710, 341.3050, 285.2787, 267.2080, 198.0530, 180.0420 |
| LysoPC(16:1) | 494.324 | 0 | 6.13 | Organic | Pos | 494.3240, 476.3140, 311.2580, 184.0730, 166.0630, 104.1070, 86.0964 |
| LysoPC(16:0) | 496.3393 | 0 | 1.23 | Aqueous | Pos | 496.3400, 478.3290, 313.2740, 258.1100, 184.0730, 166.0630,86.0964, 71.0855, 60.0808 |
| PE(20:4) | 500.2776 | 1 | 6.27 | Organic | Neg | 500.2783, 303.2330, 259.2431, 196.0380 152.9978, 140.0118 |
| LysoPE(0:0/20:3) | 504.3057 | 5 | 7.41 | Organic | Pos | 504.3080, 461.2660, 363.2890 |
| PE(20:0/0:0) | 508.3403 | 1 | 8.31 | Organic | Neg | 508.3409, 311.2956, 196.0380, 152.9958, 140.0118 |
| LysoPC(17:0) | 510.3551 | 0 | 7.05 | Organic | Pos | 510.3550, 492.3450, 327.2890, 240.1000, 184.0730, 166.0630, 104.1070, 86.0964 |
| PC(18:3) | 518.3191 | 9 | 6.66 | Organic | Pos | 518.3240, 184.0730, 86.0964, 60.0808 |
| LysoPC(18:2) | 520.3398 | 0 | 6.31 | Organic | Pos | 520.3400, 502.3290, 337.2740, 240.1000, 184.0730, 166.0630, 104.1070, 86.0964, 60.0808 |
| Lyso PC(0:0/18:1) | 522.3553 | 0 | 6.83 | Organic | Pos | 522.3550, 504.3450, 339.2890, 184.0730, 104.1070, 86.0964, 60.0808 |
| PC(0:0/18:0) | 524.371 | 0 | 7.48 | Organic | Pos | 524.3710, 506.3610, 341.3050, 285.2790, 184.0730, 104.1070, 86.0964, 60.0808 |
| PE(22:4/0:0) | 528.3091 | 0 | 6.83 | Organic | Neg | 528.3096, 331.2643, 287.2744, 214.0486, 196.0380, 140.0118, 78.9591 |
| PE(22:1/0:0) | 536.3719 | 1 | 8.33 | Organic | Pos | 536.3710, 518.3615, 395.3520, 198.0530, 62.0662 |
| PC(20:5/0:0) | 542.3217 | 4 | 6.3 | Organic | Pos | 542.3240, 483.2510, 359.2580, 184.0730, 86.0964 |
| LysoPC(20:4) | 544.3399 | 0 | 6.27 | Organic | Pos | 544.3400, 526.3290, 258.1100, 240.1000, 184.0730, 166.0630, 104.1070, 86.0964, 60.0808 |
| PS(20:1/0:0) | 550.3134 | 2 | 1.58 | Aqueous | Neg | 463.2830, 152.9958, 78.9591 |
| LysoPC(20:1/0:0) | 550.3858 | 1 | 7.6 | Organic | Pos | 550.3870, 532.3760, 367.3210, 184.0730, 104.1070, 86.0964, 60.0808 |
| PC(20:0/0:0) | 552.4024 | 0 | 8.47 | Organic | Pos | 552.4020, 293.2840, 186.0890, 184.0730, 104.1070, 86.0964 |
| PI(16:0/0:0) | 571.2881 | 1 | 6.2 | Organic | Neg | 571.2889, 315.0487, 255.2330, 241.0119, 223.0013, 171.0064, 152.9958,78.9591 |
| PS(22:1/0:0) | 578.3452 | 1 | 1.96 | Aqueous | Neg | 152.9958, 96.9591 |
| PI(18:1/0:0) | 597.3041 | 0 | 3.75 | Aqueous | Neg | 315.0487, 281.4286, 241.0119, 152.9958 |
| PI(18:0/0:0) | 599.3196 | 0 | 4.39 | Aqueous | Neg | 315.0487, 283.2643, 241.0119, 152.9958, 78.9591 |
| PE(P-16:0/18:1) | 700.5284 | 0 | 9.98 | Organic | Neg | 700.5287, 418.2728, 281.2486, 14.0118 |
| PC(P-16:0/15:1) | 702.546 | 3 | 9.95 | Organic | Pos | 702.5430, 279.2680, 184.0730 |
| SM(d18:1/16:0) | 703.574 | 1 | 9.9 | Organic | Pos | 703.5750, 184.0734, 124.9999, 86.0963 |
| SM(d18:0/16:0) | 705.5846 | 8 | 9.92 | Organic | Pos | 705.5910, 184.0730, 86.0964 |
| PE(16:0/18:1) | 716.5273 | 5 | 9.94 | Organic | Neg | 716.5236, 281.2486, 255.2330, 140.0118, 78.9591 |
| PE(P-16:0/20:4) | 722.5129 | 0 | 9.08 | Organic | Neg | 722.5130, 436.2834, 418.2728, 303.2330, 259.2431, 140.0118, 78.9591 |
| PE(0-16:0/20:4) | 724.5261 | 3 | 9.9 | Organic | Neg | 724.5287, 438.2990, 303.2330, 259.2431, 140.0118, 78.9591 |
| PE(P-18:0/18:2) | 726.5423 | 2 | 9.97 | Organic | Neg | 726.5443, 464.3147, 279.2330, 140.0118, 78.9591 |
| PC(P-16:0/17:2) | 728.5599 | 1 | 9.96 | Organic | Pos | 728.5590, 184.0730, 86.0964 |
| PC(16:1/16:0) | 732.5591 | 7 | 9.86 | Organic | Pos | 732.5540, 476.3140, 184.0730, 166.0630, 104.1070, 86.0964 |
| PC(16:0/16:0) | 734.5696 | 0 | 9.93 | Organic | Pos | 734.5690, 551.5030, 184.0730, 166.0630, 104.1070, 86.0964, 60.0802 |
| PA(0-20:0/20:4) | 737.5513 | 3 | 9.91 | Organic | Neg | 737.5191, 303.2330, 259.2431 |
| PE(18:1/18:2) | 740.5252 | 2 | 9.88 | Organic | Neg | 740.5236, 281.2486, 279.2330, 140.0118 |
| PE(18:2/18:0) | 742.5395 | 0 | 9.93 | Organic | Neg | 742.5392, 480.3096, 462.2990, 283.2643, 279.2330, 140.0118 |
| PE(18:0/18:1) | 744.5545 | 0 | 9.98 | Organic | Neg | 744.5549, 480.3096, 462.2990, 283.2643, 281.2486, 140.0118 |
| PE(P-18:0/20:4) | 750.5445 | 0 | 9.92 | Organic | Neg | 750.5443, 464.3041, 446.3041, 303.2330, 259.2431, 140.0118, 78.9591 |
| PC(18:1/16:0) | 760.585 | 0 | 9.92 | Organic | Pos | 760.5850, 184.0730, 86.0964, 60.0808 |
| PC(16:0/18:0) | 762.5954 | 6 | 9.95 | Organic | Pos | 762.6010, 184.0730, 86.0964, 60.0808 |
| PE(18:0/20:4) | 766.5413 | 2 | 9.91 | Organic | Neg | 766.5392, 480.3096, 303.2330, 283.2643, 259.2431, 140.0118 |
| PE(P-18:0/22:6) | 774.541 | 4 | 9.86 | Organic | Neg | 774.5443, 283.2431, 140.0118, 78.9591 |
| PC(14:1/22:2) | 784.5853 | 0 | 9.85 | Organic | Pos | 784.5850, 184.0730, 86.0964, 104.1070, 60.0808 |
| PC(18:1/18:1) | 786.601 | 0 | 9.92 | Organic | Pos | 786.6070, 184.0730, 166.0630, 104.0070, 86.0964, 60.0808 |
| PC(18:0/18:1) | 788.617 | 0 | 9.98 | Organic | Pos | 788.6160, 608.5500, 184.0730, 86.0964, 60.0808 |
| PS(18:1/18:0) | 790.5589 | 0 | 11.98 | Organic | Pos | 790.5590, 605.5500, 339.2890. 265.2530, 247.2420 |
| PS(0-20:0/17:1) | 790.5891 | 8 | 10.04 | Organic | Pos | 790.5690, 703.5640, 605.5870, 339.3620, 186.0160, 88.0393 |
| PC(P-20:0/18:0) | 802.6702 | 2 | 10.08 | Organic | Pos | 802.6680, 784.6580, 715.5640, 285.2790, 184.0730, 84.0808 |
| PC(16:0/22:6) | 806.573 | 4 | 9.8 | Organic | Pos | 806.5960, 788.5590, 184.0580, 86.0964, 60.0808 |
| PC(18:1/20:4) | 808.5861 | 1 | 9.83 | Organic | Pos | 808.5850, 184.0730, 104.1070, 86.0964, 60.0808 |
| PC(18:0/20:4) | 810.6026 | 2 | 9.89 | Organic | Pos | 810.6003, 184.0729, 124.9991, 60.0817 |
| PC(P-20:0/19:1) | 814.6697 | 1 | 10.04 | Organic | Pos | 814.6680, 184.0730, 86.0964 |
| PC(22:0/0-18:1) | 828.6888 | 5 | 10.06 | Organic | Pos | 828.6840, 810.6740, 184.0730, 88.1121 |
| PC(P-18:0/22:0) | 830.6973 | 2 | 10.09 | Organic | Pos | 830.7000, 184.0730, 86.0964 |
| PC(18:0/22:4) | 838.6341 | 2 | 9.94 | Organic | Pos | 838.6320, 655.5660, 186.0890, 184.0730, 88.1121 |
